# Radiomic analysis of the deltoid and scapula: identification of computed tomography-image based measurements predictive of pain, motion, and function before and after shoulder arthroplasty

**DOI:** 10.1016/j.jseint.2025.06.014

**Published:** 2025-07-05

**Authors:** Hamidreza Rajabzadeh-Oghaz, Josie Elwell, Bradley Schoch, William Aibinder, Bruno Gobbato, Daniel Wessell, Vikas Kumar, Christopher P. Roche

**Affiliations:** a Exactech, Inc., Gainesville, FL, USA; b Mayo Clinic, Florida, Jacksonville, FL, USA; c Department of Orthopedic Surgery, University of Michigan, Ann Arbor, MI, USA; d Coe Jaragua. R. José Emmendoerfer, Jaraguá do Sul, SC, Brazil

**Keywords:** Shoulder arthroplasty, Machine learning clinical outcomes predictions, Clinical decision support tools, Radiomic analysis, CT image processing, Morphological clustering analysis, Bone and muscle classifications

## Abstract

**Background:**

The goal of this study is to analyze a registry of preoperative computed tomography (CT) images of anatomic total shoulder arthroplasty (aTSA) and reverse total shoulder arthroplasty (rTSA) patients, quantify the radiomics of the deltoid muscle and scapular bone, and identify the radiomic features that are most predictive of pain, motion, and function before and after aTSA/rTSA.

**Methods:**

Preoperative CT images and clinical data from 4,009 primary shoulder arthroplasty patients were retrospectively analyzed. Next, three-dimensional masks of the deltoid (n = 2,597) and scapula (n = 3,358) were auto-segmented from CT images, and radiomic features were extracted using Py-Radiomics. These radiomics features were then used to train machine-learning regression models to predict pain, motion, and function before and after aTSA/rTSA. Finally, a clustering analysis was performed using the most predictive radiomic features to identify unique deltoid and scapula morphological groups/classes relevant to clinical outcomes before and after aTSA/rTSA.

**Results:**

Incorporating radiomic features into the machine-learning models improved the accuracy of 70.5% of deltoid model outcome predictions and 67.3% of scapular model outcome predictions. Analysis of feature importance data demonstrated that the most predictive radiomic features were numerical representations of deltoid and scapula shape and size. Notably, most shape-based radiomic features were more predictive of aTSA/rTSA outcomes than any patient demographic data (except age), comorbidity data, implant data, or diagnosis data. Finally, a radiomic-based clustering analysis identified several deltoid muscle and scapula bone morphologies associated with differences in clinical outcomes before and after aTSA/rTSA.

**Conclusion:**

This analysis of >4,000 preoperative CT scans identified numerous radiomic features of the deltoid and scapula that were highly predictive of pain, motion, and function before and after aTSA/rTSA. These most predictive radiomic features were aggregated into unique morphological clusters of deltoids and scapula that were associated with differences in clinical outcomes before and after aTSA/rTSA. Shape-based radiomic features were more predictive than first-order and second-order radiomic features, suggesting that these more interpretable measurements are more clinically relevant and could be more readily incorporated into future radiomic-based clinical decision support tools. Future work is required to further validate these radiomic findings and refine the proposed clustering analysis.

Medical images contain a large quantity of clinical data that is imperceptible to the human eye.^7^ Routine clinical interpretation of medical images is visual and qualitative; however, recent advances in artificial intelligence offer new possibilities for objective, quantitative analysis of voxel-level image data. This process of analysis, known as radiomics, transforms image data into numerical representations that can characterize a patient's three-dimensional (3D) anatomy and pathology.^13^^,^^17^^,^^18^^,^^25^ When combined with clinical data, radiomic features may help diagnosis pathology and be prognostic of treatment outcomes and complications, which is useful for guiding personalized treatment strategies.^1^^,^^9^^,^^17^^,^^18^^,^^20^^,^^25^^,^^27^^,^^29^^,^^32^^,^^35^

Over the past decade, computed tomography (CT)–based preoperative planning software has enhanced orthopedic surgeons' ability to treat numerous degenerative diseases of the shoulder with anatomic total shoulder arthroplasty (aTSA) and reverse total shoulder arthroplasty (rTSA). This planning software allows surgeons to better visualize 3D glenohumeral boney deformity and select implant size, type, and positioning that improves impingement-free range of motion and implant-to-bone surface contact. While this virtual implantation simulation is valuable, currently, no commercially available preoperative planning software provides any radiomic assessment of the bone or soft tissue in the shoulder.

Incorporating radiomic-based analyses into preoperative planning software offers the potential for a more accurate characterization of a patient's glenohumeral joint. Radiomic analyses have not yet been incorporated into this software because it is unknown which radiomic features are predictive and clinically meaningful to patients considering aTSA and rTSA. Therefore, the primary goal of this study is to analyze a large registry of preoperative CT images of aTSA and rTSA patients, quantify the radiomics of the deltoid muscle and scapular bone, and identify which radiomic features are most predictive of pain, motion, and function. A secondary goal of this study is to perform an unsupervised machine-learning (ML) clustering analysis to aggregate the most predictive radiomic features to identify unique deltoid and scapula morphological groups/classes.

## Methods

Preoperative CT images and clinical data from 4,009 primary shoulder arthroplasty patients (2,099 F, 1,910 M; age = 69.6 ± 8.4; 1,011 aTSA, 2,998 rTSA) treated with a single-platform shoulder prosthesis (Equinoxe; Exactech, Inc., Gainesville, FL, USA) were analyzed. All patients were prospectively enrolled in an institutional review board–approved multicenter study. All CT images were acquired using the ExactechGPS (Exactech, Inc., Gainesville, FL, USA) CT scan acquisition protocol, which permitted images from multiple CT scanner manufacturers using different convolution kernels. All CT images were collected within 6 months of the patient's surgery date and had slice thicknesses between 0.3 and 1.25 mm. Patients with partial acquisition of the deltoid muscle and/or scapular bone were excluded. Patients with metal artifact in the region of interest were also excluded.

Three-dimensional masks of the deltoid muscle (2,597 patients) and scapular bone (3,358 patients) were auto-segmented from preoperative CT images obtained from 38 clinical sites. Py-Radiomics (v3.0.01; Python Software Foundation, Wilmington, DE, USA) was used to extract standard radiomic features from the auto-segmented deltoid and scapula volumes. Using the built in Py-Radiomics pre-processing modules, CT images were normalized using the Z-score (to transform data to have a mean value of 0 and a standard deviation of 1), resampled to an isotropic voxel size of 1.0 × 1.0 × 1.0 mm using a Welch–Sinc interpolation, and discretized to a fixed bin count of 40. Finally, radiomic features were extracted to quantify deltoid muscle and scapular bone characteristics.

ML models were developed to predict pain, motion, and function before and after aTSA and rTSA. Predictive models were trained using 3 different sets of inputs. The study control was trained using a “minimal feature set” of inputs consisting of patient demographics, comorbidities, and preoperative planning data.^12^^,^^14^, ^15^, ^16^ A second model was trained using the control inputs and the deltoid radiomic data, and a third model was trained using the control inputs and the scapula radiomic data. XGBoost (XGBoost, Seattle, WA, USA)^4^^,^^30^ was used to train ML models that predict the American Shoulder and Elbow Surgeons (ASES), Constant, Shoulder Function, Visual Analog Scale (VAS) Pain, and the Shoulder Arthroplasty Smart (SAS)^26^ scores, as well as active abduction, forward elevation, external rotation, and the internal rotation (IR) score. Regression models predicted each measure preoperatively (except ASES and Constant) and postoperatively at 3-6 months, 6-9 months, 1-year, 2-3 years, and 3-5 years after aTSA and rTSA. For models trained with radiomic data, the relative importance of each input to predict each outcome measure preoperatively and 2-3 years postoperatively was quantified using the F-score.^2^ The F-score was averaged across all measures and rank ordered to identify the consensus top 8 radiomic features that are most predictive of pain, motion, and function before and after aTSA and rTSA.

The deltoid model utilized data from 2,597 patients (1,502 F, 1,095 M) with 4,970 postoperative visits (610 aTSA (311 F, 299 M) and 1,987 rTSA (1,191 F, 796 M)). Similarly, the scapula model utilized data from 3,358 patients (1,740 F, 1,618 M) with 6,972 postoperative visits (877 aTSA (368 F, 509 M) and 2,481 rTSA (1372F, 1109M)). This data was randomly split into 80% (training) and 20% (test) mutually exclusive datasets to train and test the predictive models at each timepoint. The process was repeated using k-fold cross validations (n = 5), dividing the dataset into 5 equal-sized subsets, to reduce overfitting. The predictive performance of each regression model was quantified by the mean absolute error.

To facilitate clinical interpretation, radiomic features were evaluated relative to (1) age and gender; (2) CT convolution kernel and gender; and (3) specific postoperative complications (deltoid muscle: instability and scapular bone: fractures), using a two-tailed unpaired t-test (*P* < .05). In addition, an unsupervised ML clustering analysis was performed using the most predictive radiomic features to identify unique deltoid and scapula morphological groups/classes. Specifically, a centroid based k-means clustering algorithm separately analyzed the deltoid and scapular cohorts using the top 8 most predictive radiomic features, along with patient age and gender. Four clusters (4 F, 4 M) for each deltoid muscle and scapular bone were defined. Preoperative and 2-year minimum clinical outcomes (deltoid: 37.9 ± 15.1 months, scapula: 37.7 ± 14.8 months) were compared to identify groups/classes associated with different outcomes using a Kruskal-Wallis test, with significance determined by (*P* < .05).

## Results

### Deltoid muscle model

The inclusion of deltoid radiomic data into the ML predictive model improved the accuracy of 110 of 156 (70.5%) regression predictions of clinical outcomes before and after aTSA and rTSA (Table I). The F-score feature importance analysis demonstrated that deltoid radiomic data was commonly used by the ML models and accounted for 17 of the top 25 features driving the preoperative model and 10 of the top 25 features driving the 2 to 3-year model (Table II). The consensus top 8 most predictive deltoid radiomic features included: normalized volume, flatness, elongation, sphericity, max two-dimensional (2D) diameter column, max 2D diameter row, fat percentage, and 10th percentile Hounsfield unit (HU). Supplementary Table S1 demonstrates that deltoid elongation, flatness, and max 2D diameter row/column measurements all differ significantly by gender across all age groups for both aTSA and rTSA patients; significant differences in deltoid fat percentage and sphericity were also observed between gender across some age groups for both aTSA and rTSA patients. Supplementary Table S2 demonstrates that deltoid volume, fat percentage, and sphericity all differ significantly between convolution kernels bone (GE) and FC30 (Toshiba) and all differ significantly between male and female patients.

**Table I:** Comparison of the MAE associated with two different ML models (deltoid muscle image data vs. no-image data) to predict clinical outcomes, preoperatively, 3 months, 6 months, 1 year, 2-3 years, and 3-5 years after aTSA and rTSA.

Clinical outcomes prediction all (aTSA, rTSA)	Preoperative MAE of predict + w/o image data (aTSA, rTSA)	Preoperative MAE of predict + with deltoid image data	Percent difference in MAE (preoperative)	3 mo MAE of predict + w/o image data (aTSA, rTSA)	3 mo MAE of predict + with deltoid image data	Percent difference in MAE (3 mo)	6 mo MAE of predict + w/o image data (aTSA, rTSA)	6 mo MAE of predict + with deltoid image data	Percent difference in MAE (6 mo)
Abduction	30.5 (30.9, 30.4)	27.2 (27.9, 26.9)	10.8% (9.6%, 11.3%)	24.2 (24.8, 24.0)	23.1 (23.9, 22.8)	4.5% (3.6%, 4.7%)	22.6 (26.0, 21.9)	21.9 (23.2, 21.7)	2.9% (11.0%, 1.0%)
External Rotation	17.4 (16.9, 17.5)	16.1 (15.1, 16.4)	7.1% (10.3%, 6.2%)	13.3 (12.8, 13.5)	13.2 (13.3, 13.2)	0.7% (−4.1%, 2.1%)	13.7 (14.3, 13.5)	13.2 (14.2, 13.0)	3.4% (0.8%, 3.9%)
Forward elevation	30.7 (28.8, 31.3)	29.0 (28.1, 29.2)	5.7% (2.4%, 6.6%)	22.5 (22.2, 22.6)	22.1 (23.4, 21.8)	1.6% (−5.8%, 3.6%)	19.8 (21.2, 19.4)	19.0 (21.2, 18.5)	4.1% (−0.2%, 4.7%)
Internal rotation score	1.5 (1.5, 1.5)	1.4 (1.3, 1.4)	6.5% (9.7%, 5.6%)	1.2 (1.2, 1.2)	1.2 (1.2, 1.2)	2.1% (0.9%, 2.5%)	1.3 (1.3, 1.3)	1.4 (1.5, 1.4)	−6.5% (−15.7%, −5.1%)
ASES	Did not model	Did not model	Did not model	13.7 (13.2, 13.9)	13.3 (12.8, 13.5)	2.9% (2.6%, 3.1%)	14.0 (13.9, 14.0)	13.9 (13.9, 13.9)	1.1% (0.3%, 1.2%)
Constant	Did not model	Did not model	Did not model	10.8 (11.5, 10.6)	10.0 (10.0, 10.0)	7.8% (13.4%, 6.4%)	9.4 (9.4, 9.4)	8.9 (8.7, 8.9)	5.8% (7.0%, 5.4%)
SAS	9.6 (9.9, 9.5)	8.9 (8.7, 8.9)	7.6% (11.8%, 6.3%)	9.3 (9.7, 9.1)	9.0 (9.9, 8.8)	2.6% (−2.1%, 3.7%)	8.4 (8.5, 8.4)	8.7 (8.9, 8.7)	−3.3% (−4.6%, −3.1%)
Global shoulder function	1.7 (1.6, 1.7)	1.6 (1.6, 1.7)	5.3% (3.7%, 5.5%)	1.6 (1.7, 1.6)	1.6 (1.6, 1.6)	2.3% (7.8%, 0.7%)	1.5 (1.4, 1.6)	1.5 (1.3, 1.6)	1.4% (5.3%, 0.6%)
VAS pain	1.9 (1.8, 1.9)	1.8 (1.7, 1.8)	5.1% (6.1%, 4.9%)	1.5 (1.4, 1.6)	1.6 (1.5, 1.6)	−1.0% (−6.2%, 0.3%)	1.6 (1.6, 1.6)	1.6 (1.5, 1.6)	1.8% (8.5%, 0.2%)

Clinical outcomes prediction all (aTSA, rTSA)	1yr MAE of predict + w/o image pata (aTSA, rTSA)	1yr MAE of predict + with deltoid image data	Percent difference in MAE (1 yr)	2-3 yr MAE of predict + w/o image data (aTSA, rTSA)	2-3 yr MAE of predict + with deltoid image data	Percent difference in MAE (2-3 yr)	3-5 yr MAE of predict + w/o image data (aTSA, rTSA)	3-5 yr MAE of predict + with deltoid image data	Percent difference in MAE (3-5 yr)
Abduction	20.7 (21.1, 20.5)	19.7 (18.8, 20.1)	4.5% (10.8%, 2.4%)	19.4 (17.7, 20.0)	18.7 (16.7, 19.3)	3.8% (5.5%, 3.6%)	18.3 (15.8, 19.3)	16.6 (15.6, 17.1)	9.3% (1.5%, 11.4%)
External rotation	12.4 (12.5, 12.3)	12.2 (12.5, 12.1)	1.1% (0.0%, 1.5%)	13.0 (12.7, 13.0)	12.9 (12.1, 13.2)	0.7% (5.2%, −0.9%)	13.0 (12.8, 13.1)	12.4 (13.7, 11.8)	4.7% (−6.6%, 9.5%)
Forward elevation	17.2 (18.3, 16.8)	16.3 (16.2, 16.4)	4.9% (11.5%, 2.7%)	15.6 (15.2, 15.8)	15.1 (15.2, 15.0)	3.5% (0.0%, 4.9%)	16.7 (14.4, 17.5)	15.5 (14.1, 16.1)	6.9% (2.1%, 8.1%)
Internal rotation score	1.2 (1.1, 1.3)	1.2 (1.1, 1.3)	−0.6% (0.5%, −0.2%)	1.2 (0.9, 1.3)	1.2 (1.0, 1.3)	−4.9% (−9.1%, −4.0%)	1.1 (1.0, 1.1)	1.1 (1.1, 1.2)	−2.7% (−6.6%, −1.4%)
ASES	12.1 (11.6, 12.2)	12.1 (11.0, 12.5)	−0.6% (5.2%, −2.2%)	12.4 (11.9, 12.6)	12.8 (11.0, 13.5)	−3.9% (7.2%, −7.4%)	11.1 (8.9, 11.9)	11.4 (9.7, 12.2)	−3.2% (−9.7%, −2.5%)
Constant	9.3 (9.6, 9.2)	9.1 (9.2, 9.1)	1.7% (3.5%, 1.2%)	9.6 (8.7, 9.8)	9.8 (9.3, 9.9)	−2.2% (−7.4%, −0.7%)	9.1 (9.6, 8.9)	8.6 (8.0, 8.9)	5.1% (16.3%, 0.5%)
SAS	8.1 (8.3, 8.0)	7.9 (7.8, 7.9)	3.0% (5.5%, 2.2%)	8.1 (7.6, 8.3)	8.0 (7.3, 8.2)	1.3% (3.8%, 0.7%)	7.5 (7.1, 7.7)	7.6 (7.8, 7.6)	−1.9% (−9.9%, 0.8%)
Global shoulder function	1.3 (1.3, 1.4)	1.3 (1.2, 1.4)	−0.1% (2.7%, −1.0%)	1.4 (1.2, 1.5)	1.4 (1.2, 1.4)	2.7% (0.2%, 3.2%)	1.4 (1.3, 1.4)	1.4 (1.3, 1.5)	−4.9% (−2.4%, −5.6%)
VAS pain	1.4 (1.4, 1.4)	1.4 (1.3, 1.5)	0.7% (8.2%, −1.8%)	1.6 (1.5, 1.6)	1.5 (1.3, 1.6)	3.1% (13.4%, −0.5%)	1.3 (1.2, 1.3)	1.3 (1.3, 1.4)	−5.1% (−9.6%, −3.6%)

*aTSA*, anatomic total shoulder arthroplasty; *rTSA*, reverse total shoulder arthroplasty; *MAE*, mean absolute error; *ASES*, American Shoulder and Elbow Surgeons; *SAS*, shoulder arthroplasty smart; *VAS*, Visual Analog Scale.

**Table II:** Comparison of the top 25 deltoid muscle model inputs (by f-score ranking) used to predict preoperative outcome measures and 2-3 year clinical outcomes after aTSA and rTSA.

Top 25 inputs for the preop & 2-3 yr postop deltoid ML models	Type of model input	Preop F-score rank	Average preop F-score across outcome models	2-3 yr F-score rank	Average 2-3 yr F-score across outcome models
Preop active abduction	Range of motion measurement	NA	NA	1	2,437.1
Glenoid retroversion	Image-based bone measurement	1	6,407.4	2	1,832.7
Preop active external rotation	Range of motion measurement	NA	NA	3	1,776.3
Preop active forward elevation	Range of motion measurement	NA	NA	4	1775.7
Preop SAS score	Patient-reported outcome score	NA	NA	5	1,638.0
Patient age	Patient demographics	4	4,427.7	6	1,631.0
Preop composite ROM score	Range of motion measurement	NA	NA	7	1,604.9
Glenoid height	Image-based bone measurement	5	4,254.1	8	1,455.0
Glenoid width	Image-based bone measurement	2	4,754.6	9	1,446.9
Normalized deltoid volume	Radiomic/image measurement	7	3,787.7	10	1,416.2
Preop pain on a daily basis	Patient-reported outcome score	NA	NA	11	1,292.6
Deltoid flatness	Radiomic measurement, shape	10	3,280.6	12	1,276.7
Preop shoulder function score	Patient-reported outcome score	NA	NA	13	1,226.7
Deltoid elongation	Radiomic measurement, shape	9	3,400.6	14	1,215.8
Preop internal rotation score	Range of motion measurement	NA	NA	15	1,118.2
Beta angle	Image-based bone measurement	3	4,605.4	16	1,111.3
Deltoid sphericity	Radiomic measurement, shape	13	2,587.9	17	1,049.3
Humeral head subluxation	Image-based joint measurement	6	3,845.3	18	1,049.2
Deltoid max 2D diameter, column	Radiomic measurement, shape	12	2,668.0	19	1,025.1
Deltoid max 2D diameter, row	Radiomic measurement, shape	11	2,765.9	20	1,023.7
Deltoid fat percentage	Radiomic/image measurement	8	3,555.6	21	1,010.4
Deltoid 10th percentile HU	Radiomic measurement, 1st order	16	2,344.3	22	926.7
Deltoid major axis length	Radiomic measurement, shape	15	2,394.6	23	906.6
Deltoid maximum	Radiomic measurement, 1st order	20	2,165.3	24	893.8
BMI	Patient demographics	19	2,199.8	25	878.2
Deltoid least axis length	Radiomic measurement, shape	14	2,544.9	27	852.7
Pixel spacing	Radiomic/image measurement	18	2,240.6	29	849.3
Deltoid skewness	Radiomic measurement, 1st order	22	2,053.4	30	833.2
Deltoid max 2D dameter, slice	Radiomic measurement, shape	17	2,271.9	31	814.9
Deltoid interquartile range	Radiomic measurement, 1st order	23	2,033.6	32	804.6
Deltoid GLCM MCC	Radiomic measurement, 2nd order	21	2,100.0	34	753.7
Was surgery on dominant hand?	Patient demographics	25	2,009.3	35	749.7
Deltoid GLSZM size zone nonuniformity normalized	Radiomic measurement, 2nd order	24	2,032.4	37	722.0

*aTSA*, anatomic total shoulder arthroplasty; *rTSA*, reverse total shoulder arthroplasty; *SAS*, shoulder arthroplasty smart score; *BMI*, body mass index; *HU*, Hounsfield unit; *ROM*, range of motion; *GLCM*, gray level co-occurrence matrix; *MCC*, maximum correlation coefficient.

To assess the clinical relevance of these radiomic findings, the deltoid radiomic features associated with rTSA patients who experienced postoperative instability (n = 24) were compared to rTSA patients without instability (n = 2,980). As described in Table III, rTSA patients with instability had comparatively larger, longer, wider, and thicker deltoids preoperatively, as demonstrated by the significantly larger values of deltoid-normalized volume (*P* = .016), least axis length (*P* = .0024), max 2D diameter column (*P* = .009), max 2D diameter row (*P* = .001), and a significantly lower deltoid surface-to-volume ratio (*P* = .001) as compared to rTSA patients without instability.

**Table III:** Comparison of average deltoid muscle radiomic measurements associated with primary rTSA patients with and without adverse event reports of instability.

Instability cohorts	Deltoid normalized volume	Deltoid elongation	Deltoid flatness	Deltoid least axis length	Deltoid major axis length	Deltoid max 2D diameter, column	Deltoid max 2D diameter, row	Deltoid mesh volume (cm^3^)	Deltoid sphericity	Deltoid surface to volume ratio	Deltoid fat percentage	Deltoid 10th percentile HU, 1st order
Stable rTSA patients	1.00 ± 0.19	0.799 ± 0.071	0.469 ± 0.045	68.2 ± 8.6	145.4 ± 12.7	160.0 ± 16.2	133.6 ± 20.2	320.2 ± 106.2	0.454 ± 0.028	0.160 ± 0.023	15.0 ± 11.5%	0.823 ± 0.159
Unstable rTSA patients	1.13 ± 0.21	0.821 ± 0.070	0.487 ± 0.039	76.0 ± 8.3	156.3 ± 13.9	172.7 ± 14.0	153.6 ± 19.8	442.9 ± 116.8	0.468 ± 0.028	0.138 ± 0.017	18.3 ± 11.5%	0.823 ± 0.136
*P* value	**.016**	.321	.176	**.002**	**.005**	**.009**	**.001**	**<.001**	.089	**.001**	.349	.987

*aTSA*, anatomic total shoulder arthroplasty; *rTSA*, reverse total shoulder arthroplasty; *2D*, two-dimensional; *GLSZM*, gray level size zone matrix.

Bold denotes statistical significance *P* < .05.

**Figure 1:**
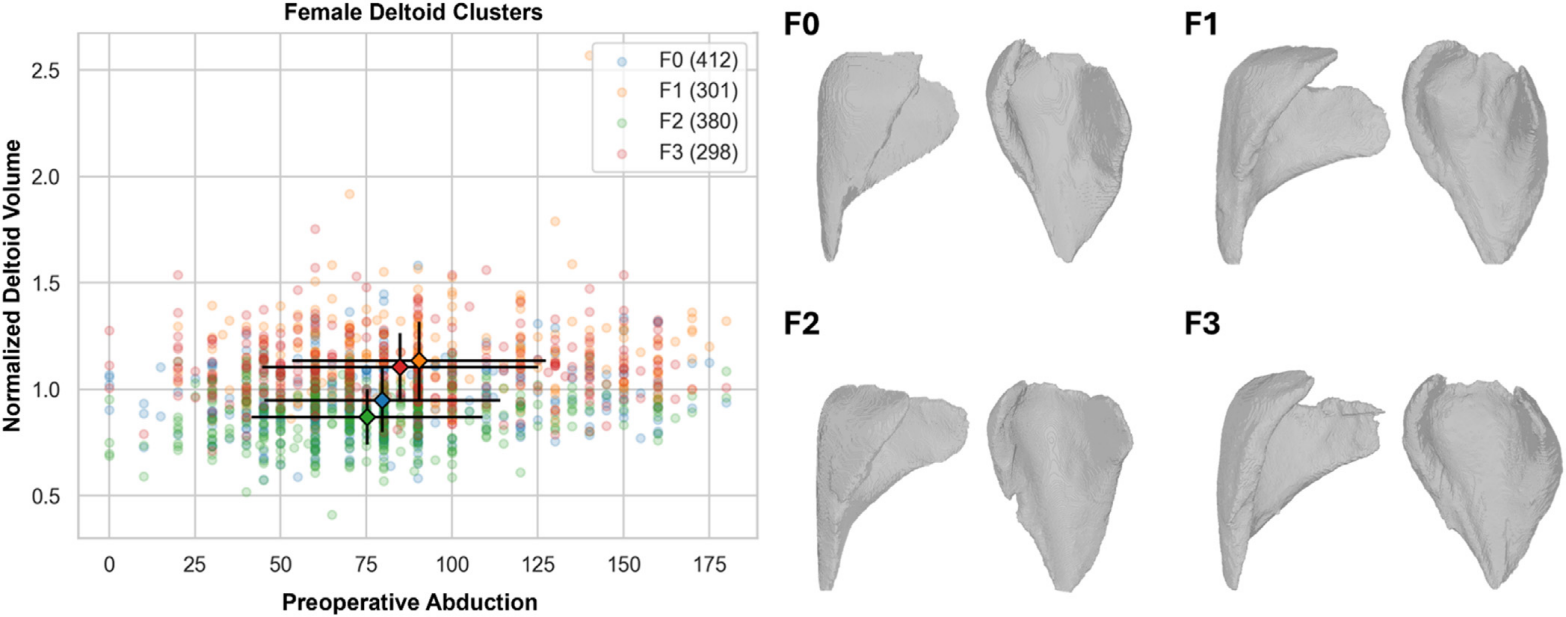
Scatter plot of preoperative abduction vs. deltoid volume normalized by age and gender (left), with corresponding 3D CT reconstructed deltoids representative (ie, near) of the centroid of each of the 4 female deltoid cluster cohorts. Note that female deltoid cluster F1 is associated with significantly more preoperative abduction than female deltoid cluster F2, as shown in Supplementary Table S4. *3D*, three-dimensional.

**Figure 2:**
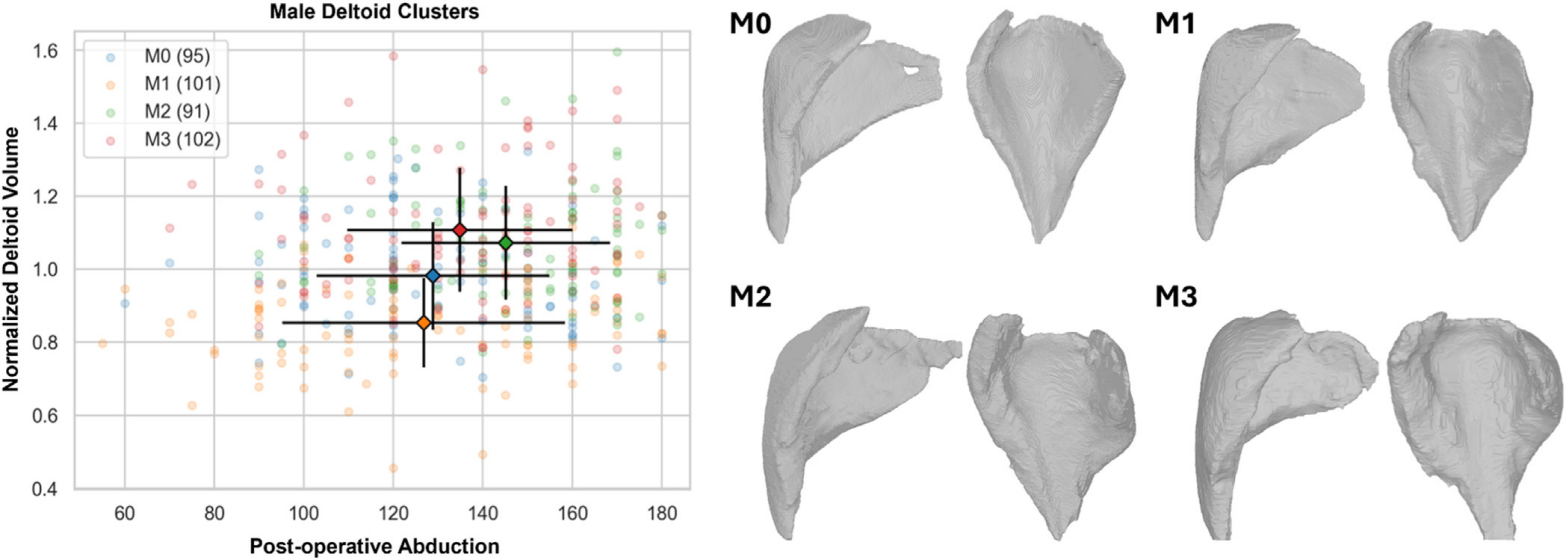
Scatter plot of 2-year minimum abduction vs. deltoid volume normalized by age and gender (left), with corresponding 3D CT reconstructed deltoids representative (ie, near) of the centroid of each of the 4 male deltoid cluster cohorts. Note that male deltoid cluster M2 is associated with significantly more 2-year minimum abduction than male deltoid cluster M1, as shown in Supplementary Table S5. *3D*, three-dimensional.

The aforementioned top 8 most predictive deltoid radiomic features were combined with patient gender and age using a cluster analysis to create 4 female and 4 male deltoid-muscle groups/classes. Distribution of radiomics between the 4 deltoid muscle cluster groups/classes for male and female patients are described in Supplementary Table S3. Supplementary Table S4 demonstrates significant differences between at least two deltoid muscle clusters for male patients in abduction, forward elevation, IR score, Constant score, and SAS score prior to surgery. Similarly, deltoid muscle clusters for female patients were identified with significant differences in abduction (Fig. 1), forward elevation, IR score, external rotation, VAS pain score, global shoulder function score, Constant score, ASES score, and SAS score prior to surgery. As described in Supplementary Table S5, deltoid muscle clusters for male patients were identified with significantly better/worse abduction (Fig. 2), IR score, and external rotation at 2-year minimum follow-up. Similarly, deltoid muscle clusters for female patients were identified with significantly better/worse forward elevation, IR score, global shoulder function score, Constant score, ASES score, and SAS score at 2-year minimum follow-up.

### Scapular bone model

The inclusion of scapula radiomic data into the ML predictive model improved the accuracy of 105 of 156 (67.3%) regression predictions of clinical outcomes before and after aTSA and rTSA (Table IV). The F-score feature importance analysis demonstrated that scapula radiomic data was commonly used by the ML models and accounted for 14 of the top 25 features driving the preoperative model and 8 of the top 25 features driving the 2 to 3-year model (Table V). The consensus top 8 most predictive scapular radiomic features included: flatness, elongation, sphericity, max 2D diameter column, max 2D diameter slice, least axis length, 10th percentile HU, and max 2D diameter row. Supplementary Table S6 demonstrates these most predictive scapular measurements differ significantly by gender across different age groups. Specifically, scapular measurements of 2D diameters column/row/slice and least axis length differed significantly by gender across all age groups for both aTSA and rTSA patients. Scapular measurements of elongation, flatness, sphericity, and 10th percentile HU differed significantly by gender for rTSA patients ≥60 years, except for the sphericity measurement of rTSA patients between 70-80 yrs. Supplementary Table S7 demonstrates that scapular 10th percentile HU and max 2D diameter slice differ significantly between Bone and FC30 kernels and male and female patients.

**Table IV:** Comparison of the MAE associated with two different machine learning models (scapular bone image data vs. no-image data) to predict clinical outcomes, preoperatively, 3 months, 6 months, 1 year, 2-3 years, and 3-5 years after aTSA and rTSA.

Clinical outcomes prediction all (aTSA, rTSA)	Preoperative MAE of predict + w/o image data (aTSA, rTSA)	Preoperative MAE of predict + with scapular bone image data	Percent difference in MAE (preoperative)	3 mo MAE of predict + w/o image data (aTSA, rTSA)	3 mo MAE of predict + with scapular bone image data	Percent difference in MAE (3 mo)	6 mo MAE of predict + w/o image data (aTSA, rTSA)	6 mo MAE of predict + with scapular bone image data	Percent difference in MAE (6 mo)
Abduction	30.5 (30.9, 30.4)	28.4 (28.8, 28.3)	6.9% (6.7%, 6.9%)	24.2 (24.8, 24.0)	22.7 (24.0, 22.2)	6.0% (3.4%, 7.2%)	22.6 (26.0, 21.9)	20.4 (21.0, 20.2)	9.7% (19.4%, 8.0%)
External rotation	17.4 (16.9, 17.5)	16.5 (15.3, 16.8)	5.4% (9.1%, 3.9%)	13.3 (12.8, 13.5)	13.5 (13.2, 13.6)	−1.3% (−3.8%, −0.8%)	13.7 (14.3, 13.5)	14.0 (14.7, 13.8)	−2.4% (−3.0%, −2.0%)
Forward elevation	30.7 (28.8, 31.3)	28.1 (26.8, 28.5)	8.6% (7.0%, 8.8%)	22.5 (22.2, 22.6)	21.8 (20.8, 22.1)	3.1% (6.2%, 2.3%)	19.8 (21.2, 19.4)	18.6 (19.2, 18.5)	5.9% (9.4%, 4.7%)
Internal rotation score	1.5 (1.5, 1.5)	1.4 (1.3, 1.4)	6.9% (9.2%, 6.0%)	1.2 (1.2, 1.2)	1.2 (1.1, 1.2)	0.0% (5.2%, −1.8%)	1.3 (1.3, 1.3)	1.3 (1.3, 1.3)	0.6% (0.4%, 0.7%)
ASES	Did not model	Did not model	Did not model	13.7 (13.2, 13.9)	13.6 (13.3, 13.7)	0.8% (−0.6%, 1.1%)	14.0 (13.9, 14.0)	13.3 (14.0, 13.1)	5.3% (−0.8%, 6.5%)
Constant	Did not model	Did not model	Did not model	10.8 (11.5, 10.6)	10.0 (10.0, 10.0)	7.5% (13.3%, 6.0%)	9.4 (9.4, 9.4)	9.3 (8.5, 9.5)	1.5% (9.5%, −0.5%)
SAS	9.6 (9.9, 9.5)	9.3 (9.1, 9.4)	3.0% (8.2%, 1.2%)	9.3 (9.7, 9.1)	8.9 (9.0, 8.9)	3.5% (7.7%, 2.2%)	8.4 (8.5, 8.4)	8.9 (8.8, 8.9)	−5.3% (−4.2%, −5.6%)
Global shoulder function	1.7 (1.6, 1.7)	1.6 (1.6, 1.7)	5.1% (2.9%, 5.2%)	1.6 (1.7, 1.6)	1.6 (1.7, 1.6)	−1.4% (−2.2%, −0.8%)	1.5 (1.4, 1.6)	1.6 (1.4, 1.6)	−0.6% (−2.1%, −1.0%)
VAS pain	1.9 (1.8, 1.9)	1.8 (1.7, 1.8)	3.1% (6.7%, 1.8%)	1.5 (1.4, 1.6)	1.6 (1.5, 1.6)	−1.9% (−5.6%, −1.4%)	1.6 (1.6, 1.6)	1.5 (1.5, 1.5)	9.0% (9.6%, 8.6%)

Clinical outcomes prediction all (aTSA, rTSA)	1yr MAE of predict + w/o image data (aTSA, rTSA)	1yr MAE of predict + with scapular bone image data	Percent difference in MAE (1 yr)	2-3 yr MAE of predict + w/o image data (aTSA, rTSA)	2-3 yr MAE of predict + with scapular bone image data	Percent difference in MAE (2-3 yr)	3-5 yr MAE of predict + w/o image data (aTSA, rTSA)	3-5 yr MAE of predict + with scapular bone image data	Percent difference in MAE (3-5 yr)
Abduction	20.7 (21.1, 20.5)	18.6 (17.9, 18.8)	10.2% (15.0%, 8.5%)	19.4 (17.7, 20.0)	18.3 (17.7, 18.6)	5.6% (−0.1%, 7.2%)	18.3 (15.8, 19.3)	18.0 (17.1, 18.3)	2.1% (−8.2%, 5.2%)
External rotation	12.4 (12.5, 12.3)	12.6 (12.9, 12.4)	−1.6% (−3.2%, −0.9%)	13.0 (12.7, 13.0)	12.4 (12.5, 12.4)	4.1% (1.7%, 4.9%)	13.0 (12.8, 13.1)	12.6 (13.7, 12.1)	2.9% (−6.9%, 7.1%)
Forward elevation	17.2 (18.3, 16.8)	15.6 (14.7, 15.9)	9.4% (20.0%, 5.6%)	15.6 (15.2, 15.8)	16.0 (15.2, 16.3)	−2.4% (0.2%, −3.3%)	16.7 (14.4, 17.5)	14.8 (13.4, 15.5)	11.1% (7.1%, 11.7%)
Internal rotation score	1.2 (1.1, 1.3)	1.2 (1.1, 1.3)	−0.1% (−0.5%, 0.0%)	1.2 (0.9, 1.3)	1.2 (1.0, 1.2)	0.8% (−9.0%, 2.2%)	1.1 (1.0, 1.1)	1.1 (1.1, 1.1)	1.9% (−8.8%, 5.4%)
ASES	12.1 (11.6, 12.2)	11.7 (11.3, 11.8)	3.2% (3.0%, 3.3%)	12.4 (11.9, 12.6)	12.4 (11.5, 12.8)	−0.2% (3.4%, −1.8%)	11.1 (8.9, 11.9)	11.2 (10.5, 11.4)	−0.7% (−18.1%,3.4%)
Constant	9.3 (9.6, 9.2)	9.0 (8.0, 9.3)	3.5% (16.2%, −0.5%)	9.6 (8.7, 9.8)	9.6 (9.5, 9.6)	−0.4% (−8.9%, 2.0%)	9.1 (9.6, 8.9)	7.9 (8.0, 7.8)	13.7% (16.0%, 12.5%)
SAS	8.1 (8.3, 8.0)	7.6 (6.8, 8.0)	5.8% (18.3%, 1.1%)	8.1 (7.6, 8.3)	7.6 (7.3, 7.7)	6.4% (3.8%, 7.2%)	7.5 (7.1, 7.7)	7.1 (6.9, 7.2)	5.6% (3.0%, 6.1%)
Global shoulder function	1.3 (1.3, 1.4)	1.3 (1.1, 1.4)	2.3% (12.5%, −0.9%)	1.4 (1.2, 1.5)	1.4 (1.2, 1.5)	−0.2% (−0.7%, −0.3%)	1.4 (1.3, 1.4)	1.4 (1.3, 1.4)	0.1% (0.0%, 0.1%)
VAS pain	1.4 (1.4, 1.4)	1.3 (1.2, 1.4)	6.5% (14.1%, 4.0%)	1.6 (1.5, 1.6)	1.4 (1.4, 1.5)	7.9% (8.1%, 7.7%)	1.3 (1.2, 1.3)	1.3 (1.2, 1.3)	−2.3% (−4.5%, −1.7%)

*aTSA*, anatomic total shoulder arthroplasty; *rTSA*, reverse total shoulder arthroplasty; *MAE*, mean absolute error; *ASES*, American Shoulder and Elbow Surgeons; *SAS*, shoulder arthroplasty smart score; *VAS*, Visual Analog Scale.

**Table V:** Comparison of the top 25 scapular bone model inputs (by F-score ranking) used to predict preoperative outcome measures and 2-3 year clinical outcomes after aTSA and rTSA.

Top 25 inputs for the preop & 2-3 yr postop scapular bone ML models	Type of model input	Preop F-score rank	Average preop F-score across outcome models	2-3 yr F-score rank	Average 2-3 yr F-score across outcome models
Preop active abduction	Range of motion measurement	NA	NA	1	3,105.7
Glenoid retroversion	Image-based bone measurement	1	7,168.4	2	2,482.3
Preop active external rotation	Range of motion measurement	NA	NA	3	2,205.1
Preop active forward elevation	Range of motion measurement	NA	NA	4	2,164.6
Patient age	Patient demographic	3	4,882.7	5	2,104.4
Preop composite ROM score	Range of motion measurement	NA	NA	6	2,030.4
Preop SAS score	Patient-reported outcome score	NA	NA	7	1,908.9
Glenoid width	Image-based bone measurement	2	5,064.1	8	1,833.6
Glenoid height	Image-based bone measurement	4	4,836.0	9	1,805.8
Scapula flatness	Radiomic measurement, shape	7	3,690.1	10	1,629.2
Scapular elongation	Radiomic measurement, shape	6	4,146.0	11	1,566.7
Preop pain on a daily basis	Patient-reported outcome score	NA	NA	12	1,547.8
Preop shoulder function score	Patient-reported outcome score	NA	NA	13	1,467.2
Scapular sphericity	Radiomic measurement, shape	13	2,839.0	14	1,431.8
Scapula max 2D diameter, column	Radiomic measurement, shape	9	3,389.1	15	1,427.0
Beta angle	Image-based bone measurement	5	4,252.4	16	1,366.7
Preop internal rotation score	Range of motion measurement	NA	NA	17	1,291.9
Scapula max 2D diameter, slice	Radiomic measurement, shape	11	3,080.3	18	1,284.6
Scapula least axis length	Radiomic measurement, shape	12	3,008.0	19	1,267.2
Scapula 10th percentile HU	RadiomicmMeasurement, 1st order	15	2,749.7	20	1,247.8
BMI	Patient demographic	16	2,490.4	21	1,202.4
Humeral head subluxation	Image-based joint measurement	8	3,665.3	22	1,197.6
Scapula max 2D diameter, row	Radiomic measurement, shape	10	3,103.7	23	1,181.9
Device type	Prosthesis type (aTSA or rTSA)	NA	NA	24	1,145.9
Scapula mean	Radiomic measurement, 1st order	18	2,313.1	25	1,117.1
Scapula kurtosis	Radiomic measurement, 1st order	20	2,299.1	26	1,108.8
Was surgery on dominant hand?	Patient demographic	24	2,110.3	27	1,065.9
Scapula major axis length	Radiomic measurement, shape	14	2,822.7	28	1,061.4
Pixel spacing	Radiomic/image measurement	19	2,304.6	29	1,055.4
Scapula minimum	Radiomic measurement, 1st order	22	2,205.1	30	1,036.8
Scapula minor axis length	Radiomic measurement, shape	21	2,236.7	31	1,000.2
Scapula surface volume ratio	Radiomic measurement, shape	25	2,015.6	33	967.0

*aTSA*, anatomic total shoulder arthroplasty; *rTSA*, reverse total shoulder arthroplasty; *SAS*, shoulder arthroplasty smart score; *BMI*, body mass index; *HU*, Hounsfield unit; *ROM*, range of motion.

To assess the clinical relevance of these radiomic findings, the scapula radiomic features associated with rTSA who experienced scapular fractures (n = 28) were compared to rTSA patients without scapular fractures (n = 2,976). As described in Table VI, rTSA patients with scapular fractures had comparatively smaller, thinner, shorter, and less spherical scapula preoperatively, as demonstrated by the significantly smaller values of scapula least axis length (*P* = .004), minor axis length (*P* = .012), max 2D diameter row (*P* = .016), max 2D diameter slice (*P* = .009), mesh volume (*P* = .001), sphericity (*P* = .049), and a significantly larger scapula surface-to-volume ratio (*P* < .001) and neighborhood gray tone difference matrix Strength (*P* < .001) as compared to rTSA patients without scapular fractures.

**Table VI:** Comparison of average scapula bone radiomic measurements associated with primary rTSA patients with and without adverse event reports of scapula fractures.

Scapula fracture cohorts	Scapula elongation	Scapula flatness	Scapula least axis length (mm)	Scapula minor axis length (mm)	Scapula max 2D diameter, column (mm)	Scapula max 2D diameter, row (mm)	Scapula max 2D diameter, slice (mm)	Scapula mesh volume (cm^3^)	Scapula sphericity	Scapula surface to volume ratio	Scapula 10th percentile	Scapula NGTDM strength
Nonfx patients	0.509 ± 0.035	0.298 ± 0.028	54.0 ± 6.0	92.4 ± 8.3	114.1 ± 15.2	153.2 ± 16.9	130.9 ± 13.4	78.8 ± 25.4	0.232 ± 0.014	0.500 ± 0.069	0.978 ± 0.178	0.077 ± 0.024
Scapula fx patients	0.510 ± 0.029	0.292 ± 0.023	50.4 ± 4.4	88.0 ± 5.6	109.6 ± 14.7	144.6 ± 14.9	123.6 ± 12.1	61.3 ± 16.4	0.226 ± 0.012	0.551 ± 0.052	1.024 ± 0.167	0.096 ± 0.021
*P* value	.917	.329	**.004**	**.012**	.154	**.016**	**.009**	**.001**	**.049**	**<.001**	.219	**<.001**

*rTSA*, reverse total shoulder arthroplasty; *2D*, two-dimensional; *NGTDM*, neighborhood gray tone difference matrix.

Bold denotes statistical significance *P* < .05.

**Figure 3:**
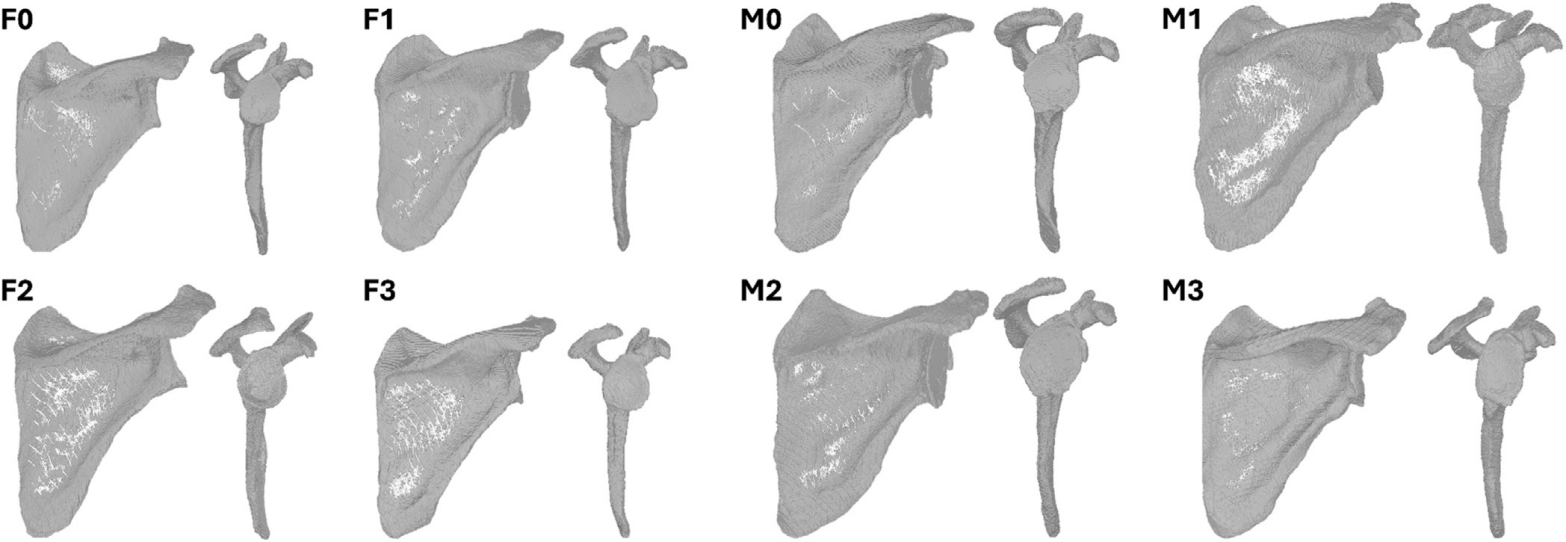
3D CT reconstructed scapula representative (ie, near) of the centroid of each of the 4 female (*left*) and 4 male (*right*) Scapula bone cluster cohorts. *3D*, three-dimensional.

The aforementioned top 8 most predictive scapular radiomic features were combined with patient gender and age using a cluster analysis to create 4 female and 4 male scapular bone groups/classes. Distribution of radiomics between the 4 scapular bone cluster groups/classes for male and female patients are described in Supplementary Table S8 and depicted in Figure 3. Supplementary Table S9 demonstrates significant differences between at least 2 scapular bone clusters for male patients in IR score, global shoulder function score, and Constant score prior to surgery. Similarly, scapular bone clusters for female patients were identified with significant differences in abduction, external rotation, VAS pain score, and ASES score prior to surgery. Finally, as described in Supplementary Table S10, scapular bone clusters for female patients were identified with significantly better/worse external rotation at 2-year minimum follow-up; however, no 2-year minimum clinical outcome differences in scapular bone clusters were observed for male patients.

## Discussion

The results of this >4,000 patient ML-based CT analysis identified numerous deltoid muscle and scapular bone radiomic features that are highly predictive of pain, motion, and function before and after aTSA and rTSA. The most clinically relevant and predictive radiomic features were numerical representations of deltoid and scapula shape and size; however, some first-order and second-order radiomic features were also found to be predictive. Notably, these top-ranked radiomic features were more predictive of aTSA/rTSA outcomes than any patient demographic data (except age), comorbidity data, implant data, or diagnosis data. In addition, a novel clustering analysis utilizing the most predictive radiomic features identified several deltoid and scapula morphologies associated with differences in clinical outcomes before and after aTSA/rTSA. Cumulatively, these findings demonstrate the potential of CT image–based radiomic measurements, when incorporated in ML-based clinical decision support tools (CDSTs), to facilitate more personalized treatment decision-making for clinicians and patients considering shoulder arthroplasty.

Differences in radiomic measurements of the deltoid muscle and scapular bone were observed between gender, age, and CT convolution kernels for both aTSA and rTSA patients. Specifically, deltoid radiomic measurements of elongation, flatness, and max 2D diameter row/column and scapular radiomic measurements of max 2D diameter column/row/slice and least axis length all differed significantly by gender across all age groups analyzed for both aTSA and rTSA patients. In addition, deltoid radiomic measurements of normalized volume, elongation, fat percentage, sphericity, and 10th percentile HU and scapular radiomic measurements of sphericity, max 2D diameter column/slice, and 10th percentile HU all differed significantly between two commonly utilized CT convolution kernels. Differences in radiomic measurements were also observed between rTSA patients with instability (vs. patients without instability) and between rTSA patients with scapular fractures (vs. patients without fractures). While additional clinical validation is required, the use of our ML framework to automatically extract radiomic measurements from preoperative CT images that are highly predictive of aTSA and rTSA clinical outcomes is promising and exciting; these image-based ML techniques offer multiple new possibilities for clinical analysis and orthopedic research and have potential to augment treatment guidelines when incorporated into novel ML-based CDSTs.

Medical images contain a large quantity of data that is not routinely analyzed in clinical practice. Traditional interpretation of medical imaging is visual and subjective; radiomic analysis provides a new method for more objective and granular quantification of image data. To illustrate the quantity of data available in a typical shoulder CT scan, a 512 × 512 CT image contains 262,144 pixels, and nearly 350 image slices, resulting in ∼ 92 million pixels per patient scan. Given that this study utilized CT scans from nearly 4,000 patients, our radiomic analysis considered >365 billion pixels. Such a large quantity of data could only be analyzed with the aid of artificial intelligence. Moreover, the radiomic measurements analyzed in this study, which are numerical representations of a region of interest in the medical images, could only be calculated with the aid of computers. Specifically in our study, we identified that the most predictive deltoid radiomic features were normalized volume, flatness, and elongation and the most predictive scapular radiomic features were flatness, elongation, and sphericity. These radiomic features mathematically describe different aspects of the size and shape of each segmented region of interest. For example, elongation describes the relationship between the two largest principal components, with a range of values range between 1 (where the cross-section through the first and second largest principal moments is circle-like (nonelongated)) and 0 (where the object is a maximally elongated: ie, a 1-dimensional line); flatness describes the relationship between the largest and smallest principal components, with a range of values between 1 (nonflat, sphere-like) and 0 (a flat object, or single-slice segmentation); and sphericity describes the roundness of the region of interest relative to a sphere, with a range of values between 0 and 1 (perfect sphere). For additional information, please refer to the standard definitions for Py-Radiomics radiomic features in Supplementary Table S11.

Using an ML-based framework, we automatically analyzed standard-of-care, real-world CT images from multiple scanners and identified a diverse set of radiomic features that are the most clinically relevant and predictive of outcomes before and after aTSA and rTSA, which is a crucial step towards development of an ML-based CDST preoperative planning software. One barrier to the adoption of radiomic-based ML-CDSTs is the need for clinicians to understand the basis of those predictions. The clinical interpretability of CDSTs utilizing shape-based radiomic inputs is more intuitive than CDSTs utilizing HU-intensity and texture inputs, since these first-order and second-order radiomic features are voxel-level measurements that are imperceptible to the naked eye. It is noteworthy that deltoid and scapula shape-based radiomic features were associated with higher F-score rankings than first-order and second-order radiomic features. When these radiomic measurements are eventually deployed in a CDST, the use of a graphical user interface to display the axes/components of each region of interest may help the surgeon-user better visualize and interpret how these radiomic measurements are calculated.

Clustering is an unsupervised ML-based approach to classify complex datasets into distinct groups having unique characteristics. This dimensionality-reduction technique can improve interpretation of radiomic analyses by consolidating numerous voxel-level measurements into morphologically distinct groups that can be more readily visualized. Moreover, this method of aggregating the most predictive radiomic features occurring naturally together in a study population could be a useful technique to create clinically relevant classification systems of different anatomical/morphological structures. Notably in our study, a few muscle/bone clusters were associated with significantly more/less motion, function, and pain before/after aTSA/rTSA. Future work is necessary to refine/optimize the clustering analyses for different outcome measures and/or complication risks and assess if these conglomerate clusters are more predictive (ie, have greater feature importance) of aTSA/rTSA outcomes than the constituent radiomic features. However, once bone and muscle clusters have been clinically validated, these clusters can be readily incorporated into preoperative planning software and used prospectively to classify the bones and muscles of an individual patient to further characterize a patient's joint and refine diagnosis, further personalize treatment decision-making, and further improve the accuracy of clinical outcomes predictions.

This study is the first of its kind to analyze radiomic features of bones and muscles in the shoulder and use that image-based data to predict pain, motion, and function before and after aTSA and rTSA. It is also the largest study to use image-based measurements to predict clinical outcomes. Nearly all the existing clinical literature analyzing images of the shoulder have limited generalizability and limited statistical power due to small sample sizes (n ≤ 100).^8^^,^^10^^,^^22^, ^23^, ^24^^,^^31^^,^^34^ In addition, these studies have limited comparative validity due to limitations in their methodology, as these studies were generally performed with 2D measurements instead of 3D, and with manual measurements instead of automated. More importantly, these studies often failed to adequately report the raw image data characteristics and the image-processing techniques used.^8^^,^^10^^,^^22^, ^23^, ^24^^,^^31^^,^^34^ Radiomic-based studies require strict guidelines for reporting, including which radiomics software/version was used, imaging protocols (especially convolution kernels), and image-processing steps (normalization, resampling, discretization), to facilitate reproducibility and comparison. Future work is required to standardize methodologies throughout the radiomic process.^6^^,^^33^^,^^36^

This study has numerous limitations. First, we utilized CT image data from 38 clinical sites, which provided images from different CT scanner manufacturers using a range of image acquisition protocols (eg, slice thickness and kernels). While the inclusion of real-world CT images improves overall study generalizability, it adds substantial variability. To mitigate this, we performed several image-processing steps to normalize the images; however, some differences remain, particularly between measurements obtained from Bone and FC30 kernels. As an additional mitigation, CT kernel, slice thickness, and pixel spacing were included as ML model inputs. Second, our radiomic analysis utilized standard Py-Radiomics features, which have been previously utilized for oncology applications and have thus far had limited applications in the shoulder. Future work should consider additional radiomic features and evaluate other image-based techniques, like embeddings. Third, it is known that radiomic features are affected by image acquisition/processing variables^5^^,^^6^^,^^11^^,^^19^^,^^21^; as such, a sensitivity assessment is required to ensure that all radiomic measures are robust/stable and unique across the range of CT image acquisition/processing steps. Fourth, we separately analyzed deltoid and scapula radiomics and did not attempt to combine the deltoid and scapular radiomic data into a single predictive model because of the large number of features. Future work should train a combined radiomic model consisting of only the most predictive features to reduce the risk of overfitting. Fifth, we analyzed the overall deltoid and scapula and did not attempt to individually characterize any individual deltoid segment (anterior, middle, posterior heads) or any specific portion of the scapula, like the glenoid vault, acromion, or scapular spine. Focused radiomic analysis of targeted bone segments may improve predictions of complications like aseptic glenoid loosening or acromial/scapular fractures. Sixth, we did not analyze the rotator cuff, pectoralis, latissimus, or scapular elevator muscles, nor did we analyze the humerus or clavicle bones. Future work should analyze these other relevant glenohumeral muscles and bones to better understand the contribution of each on aTSA/rTSA clinical outcomes and complications. Seventh, we did not conduct a fairness assessment of our radiomic-based ML predictions, like Allen et al^3^, nor did we perform an external validation, like Simmons et al.^28^ Future work is required to analyze the fairness and bias associated with predictions derived using radiomic inputs, and future work is required to externally validate these findings. Eighth, our ML models, both nonimage and image-based, were developed from a dataset of primary aTSA and primary rTSA patients using one-platform shoulder prosthesis, where patients with revisions, humeral fractures, or hemiarthroplasty were excluded. As such, model predictions may not be translatable for those excluded indications or other prosthesis types or designs. Finally, our ML analysis utilized retrospective clinical data to identify numerous features that were highly predictive of pain, motion, and function before and after aTSA and rTSA. Given this use of retrospective data, the predictive power of these identified radiomic features is assumed to be correlative and not causative/deterministic.

## Conclusion

Our ML framework successfully analyzed preoperative CT images from >4,000 patients, quantified radiomics of the deltoid muscle and scapula bone, and identified the specific radiomic features that were most predictive of clinical outcomes before and after aTSA and rTSA. Incorporating these radiomic features into our ML models improved the accuracy of pain, motion, and function predictions before and after aTSA and rTSA. Supplementing this image-based ML analysis with a clustering analysis demonstrated how radiomic features can be aggregated to create novel, clinically relevant classification systems—which may improve interpretability of these voxel-level measurements while also helping physicians better understand the relationship between a patient's anatomy and physiology, and better understand a patient’s potential for clinical improvement after treatment with aTSA and rTSA. Future work is necessary to clinically validate this ML framework and better understand the association between each radiomic measurement (and each aggregate cluster) and each functional outcome before and after aTSA/rTSA.

## Disclaimers:

Funding: No funding was provided to complete this study; however, Exactech Inc. (Gainesville, FL) funded data collection for the clinical data used in this study. No authors have any stock or stock options in Exactech, Inc.

Conflicts of interest: Bradley Schoch, William Aibinder, Bruno Gobbato, and Daniel Wessell are consultants for Exactech, Inc. Hamidreza Rajabzadeh-Oghaz, Josie Elwell, Vikas Kumar, and Christopher Roche are employed by Exactech, Inc. The other authors, their immediate families, and any research foundations with which they are affiliated have not received any financial payments or other benefits from any commercial entity related to the subject of this article.

## References

[1] Aerts H.J., Velazquez E.R., Leijenaar R.T., Parmar C., Grossmann P., Carvalho S. (2014). Decoding tumour phenotype by noninvasive imaging using a quantitative radiomics approach. Nat Commun.

[2] Ahmad M.A., Eckert C., Teredesai A. (2018). Interpretable machine learning in healthcare. IEEE Intell Inform Bull.

[3] Allen C., Kumar V., Elwell J., Overman S., Schoch B.S., Aibinder W. (2024). Evaluating the fairness and accuracy of machine learning-based predictions of clinical outcomes after anatomic and reverse total shoulder arthroplasty. J Shoulder Elbow Surg.

[4] Chen T., Guestrin C. (2016). Proceedings of the 22nd ACM SIGKDD international conference on knowledge discovery and data mining.

[5] Escudero Sanchez L., Rundo L., Gill A.B., Hoare M., Mendes Serrao E., Sala E. (2021). Robustness of radiomic features in CT images with different slice thickness, comparing liver tumour and muscle. Sci Rep.

[6] Fornacon-Wood I., Mistry H., Ackermann C.J., Blackhall F., McPartlin A., Faivre-Finn C. (2020). Reliability and prognostic value of radiomic features are highly dependent on choice of feature extraction platform. Eur Radiol.

[7] Gillies R.J., Kinahan P.E., Hricak H. (2016). Radiomics: images are more than pictures, they are data. Radiology.

[8] Holzbaur K.R., Murray W.M., Gold G.E., Delp S.L. (2007). Upper limb muscle volumes in adult subjects. J Biomech.

[9] Jiang Y.W., Xu X.J., Wang R., Chen C.M. (2022). Radiomics analysis based on lumbar spine CT to detect osteoporosis. Eur Radiol.

[10] Kälin P.S., Crawford R.J., Marcon M., Manoliu A., Bouaicha S., Fischer M.A. (2018). Shoulder muscle volume and fat content in healthy adult volunteers: quantification with DIXON MRI to determine the influence of demographics and handedness. Skeletal Radiol.

[11] Koçak B., Yüzkan S., Mutlu S., Karagülle M., Kala A., Kadıoğlu M. (2024). Influence of image preprocessing on the segmentation-based reproducibility of radiomic features: in vivo experiments on discretization and resampling parameters. Diagn Interv Radiol.

[12] Kumar V., Allen C., Overman S., Teredesai A., Simovitch R., Flurin P.H. (2022). Development of a predictive model for a machine learning–derived shoulder arthroplasty clinical outcome score. Semin Arthroplasty.

[13] Kumar V., Gu Y., Basu S., Berglund A., Eschrich S.A., Schabath M.B. (2012). Radiomics: the process and the challenges. Magn Reson Imaging.

[14] Kumar V., Roche C., Overman S., Simovitch R., Flurin P.-H., Wright T. (2021). Using machine learning to predict clinical outcomes after shoulder arthroplasty with a minimal feature set. J Shoulder Elbow Surg.

[15] Kumar V., Roche C., Overman S., Simovitch R., Flurin P.-H., Wright T. (2020). What is the accuracy of three different machine learning techniques to predict clinical outcomes after shoulder arthroplasty?. Clin Orthopaedics Relat Res.

[16] Kumar V., Schoch B.S., Allen C., Overman S., Teredesai A., Aibinder W. (2022). Using machine learning to predict internal rotation after anatomic and reverse total shoulder arthroplasty. J Shoulder Elbow Surg.

[17] Lambin P., Leijenaar R.T.H., Deist T.M., Peerlings J., de Jong E.E.C., van Timmeren J. (2017). Radiomics: the bridge between medical imaging and personalized medicine. Nat Rev Clin Oncol.

[18] Lambin P., Rios-Velazquez E., Leijenaar R., Carvalho S., van Stiphout R.G., Granton P. (2012). Radiomics: extracting more information from medical images using advanced feature analysis. Eur J Cancer.

[19] Ligero M., Jordi-Ollero O., Bernatowicz K., Garcia-Ruiz A., Delgado-Muñoz E., Leiva D. (2021). Minimizing acquisition-related radiomics variability by image resampling and batch effect correction to allow for large-scale data analysis. Eur Radiol.

[20] Louis T., Lucia F., Cousin F., Mievis C., Jansen N., Duysinx B. (2024). Identification of CT radiomic features robust to acquisition and segmentation variations for improved prediction of radiotherapy-treated lung cancer patient recurrence. Sci Rep.

[21] Lu L., Ehmke R.C., Schwartz L.H., Zhao B. (2016). Assessing agreement between radiomic features computed for multiple CT imaging settings. PLoS One.

[22] McClatchy S.G., Heise G.M., Mihalko W.M., Azar F.M., Smith R.A., Witte D.H. (2022). Effect of deltoid volume on range of motion and patient-reported outcomes following reverse total shoulder arthroplasty in rotator cuff-intact and rotator cuff-deficient conditions. Shoulder Elbow.

[23] Meyer D.C., Rahm S., Farshad M., Lajtai G., Wieser K. (2013). Deltoid muscle shape analysis with magnetic resonance imaging in patients with chronic rotator cuff tears. BMC Musculoskelet Disord.

[24] Nakazawa K., Manaka T., Hirakawa Y., Ito Y., Iio R., Oi N. (2023). Reliability and validity of a new deltoid muscle area measurement method after reverse shoulder arthroplasty. JSES Int.

[25] Rajabzadeh-Oghaz H., Kumar V., Berry D.B., Singh A., Schoch B.S., Aibinder W.R. (2024). Impact of deltoid computer tomography image data on the accuracy of machine learning predictions of clinical outcomes after anatomic and reverse total shoulder arthroplasty. J Clin Med.

[26] Roche C., Kumar V., Overman S., Simovitch R., Flurin P.-H., Wright T. (2021). Validation of a machine learning-derived clinical metric to quantify outcomes after total shoulder arthroplasty. J Shoulder Elbow Surg.

[27] Rogers W., Thulasi Seetha S., Refaee T.A.G., Lieverse R.I.Y., Granzier R.W.Y., Ibrahim A. (2020). Radiomics: from qualitative to quantitative imaging. Br J Radiol.

[28] Simmons C., DeGrasse J., Polakovic S., Aibinder W., Throckmorton T., Noerdlinger M. (2023). Initial clinical experience with a predictive clinical decision support tool for anatomic and reverse total shoulder arthroplasty. Eur J Orthop Surg Traumatol.

[29] Sugai Y., Kadoya N., Tanaka S., Tanabe S., Umeda M., Yamamoto T. (2021). Impact of feature selection methods and subgroup factors on prognostic analysis with CT-based radiomics in non-small cell lung cancer patients. Radiat Oncol.

[30] Torlay L., Perrone-Bertolotti M., Thomas E., Baciu M. (2017). Machine learning–XGBoost analysis of language networks to classify patients with epilepsy. Brain Inform.

[31] Vidt M.E., Daly M., Miller M.E., Davis C.C., Marsh A.P., Saul K.R. (2012). Characterizing upper limb muscle volume and strength in older adults: a comparison with young adults. J Biomech.

[32] Vogele D., Mueller T., Wolf D., Otto S., Manoj S., Goetz M. (2024). Applicability of the CT radiomics of skeletal muscle and machine learning for the detection of sarcopenia and prognostic assessment of disease progression in patients with gastric and esophageal tumors. Diagnostics (Basel).

[33] Whybra P., Zwanenburg A., Andrearczyk V., Schaer R., Apte A.P., Ayotte A. (2024). The image biomarker standardization initiative: standardized convolutional filters for reproducible radiomics and enhanced clinical insights. Radiology.

[34] Wiater B.P., Koueiter D.M., Maerz T., Moravek J.E., Yonan S., Marcantonio D.R. (2015). Preoperative deltoid size and fatty infiltration of the deltoid and rotator cuff correlate to outcomes after reverse total shoulder arthroplasty. Clin Orthop Relat Res.

[35] Yang L., Yang J., Zhou X., Huang L., Zhao W., Wang T. (2019). Development of a radiomics nomogram based on the 2D and 3D CT features to predict the survival of non-small cell lung cancer patients. Eur Radiol.

[36] Zwanenburg A., Vallières M., Abdalah M.A., Aerts H.J.W.L., Andrearczyk V., Apte A. (2020). The image biomarker standardization initiative: standardized quantitative radiomics for high-throughput image-based phenotyping. Radiology.

