# Supplementary material for: Radiomic analysis of the deltoid and scapula: identification of computed tomography-image based measurements predictive of pain, motion, and function before and after shoulder arthroplasty
Source: JSES Int. 2025 Jul 5;9(6):2087–97. doi: 10.1016/j.jseint.2025.06.014 (PMC12828216; doi:10.1016/j.jseint.2025.06.014)
Supplement: Supplementary Table [file mmc1.docx]

**Supplemental Table 1**. Comparison of average deltoid muscle radiomic measurements associated with primary aTSA and rTSA patients, stratified by gender and age at the time of surgery

| **aTSA, rTSA** | **Age at time of surgery** | **<60 yrs** | **60-70 yrs** | **70-80 yrs** |
| --- | --- | --- | --- | --- |
| **Deltoid Normalized Volume** | Male | 1.05 ± 0.21, 0.95 ± 0.20 | 1.03 ± 0.19, 0.99 ± 0.19 | 1.01 ± 0.18, 1.00 ± 0.18 |
|  | Female | 1.01 ± 0.17, 1.01 ± 0.18 | 1.00 ± 0.18, 1.00 ± 0.20 | 1.00 ± 0.17, 1.00 ± 0.19 |
|  | *p value* (aTSA, rTSA) | 0.2653, 0.1140 | 0.1166, 0.7608 | 0.5870, 0.7628 |
| **Deltoid Elongation** | Male | 0.854 ± 0.061, 0.852 ± 0.059 | 0.855 ± 0.065, 0.845 ± 0.065 | 0.839 ± 0.058, 0.837 ± 0.062 |
|  | Female | 0.771 ± 0.060, 0.771 ± 0.066 | 0.768 ± 0.050, 0.776 ± 0.064 | 0.766 ± 0.061, 0.772 ± 0.064 |
|  | *p value* (aTSA, rTSA) | **<0.001, <0.001** | **<0.001, <0.001** | **<0.001, <0.001** |
| **Deltoid Flatness** | Male | 0.474 ± 0.035, 0.479 ± 0.041 | 0.472 ± 0.036, 0.483 ± 0.042 | 0.473 ± 0.034, 0.486 ± 0.038 |
|  | Female | 0.455 ± 0.034, 0.451 ± 0.038 | 0.451 ± 0.036, 0.455 ± 0.044 | 0.454 ± 0.042, 0.457 ± 0.045 |
|  | *p value* (aTSA, rTSA) | **0.003, <0.001** | **<0.001, <0.001** | **0.002, <0.001** |
| **Deltoid Fat Percentage (%)** | Male | 12.6 ± 10.6, 15.7 ± 10.6 | 11.6 ± 10.4, 14.1 ± 11.3 | 11.2 ± 10.4, 13.4 ± 11.4 |
|  | Female | 16.5 ± 10.4, 17.2 ± 10.7 | 14.6 ± 10.7, 17.2 ± 11.7 | 12.3 ± 9.1, 15.5 ± 11.7 |
|  | *p value* (aTSA, rTSA) | **0.048,** 0.432 | **0.013, 0.001** | 0.429, **0.005** |
| **Deltoid Max 2D Diameter, Row (mm)** | Male | 150.5 ± 13.1, 147.2 ± 14.9 | 150.3 ± 16.0, 149.2 ± 15.8 | 147.0 ± 16.8, 148.4 ± 17.1 |
|  | Female | 121.7 ± 14.0, 124.1 ± 14.5 | 121.2 ± 15.3, 124.1 ± 16.1 | 122.8 ± 18.1, 123.3 ± 16.1 |
|  | *p value* (aTSA, rTSA) | **<0.001, <0.001** | **<0.001, <0.001** | **<0.001, <0.001** |
| **Deltoid Sphericity** | Male | 0.476 ± 0.026, 0.467 ± 0.025 | 0.462 ± 0.027, 0.465 ± 0.028 | 0.451 ± 0.024, 0.451 ± 0.024 |
|  | Female | 0.469 ± 0.028, 0.468 ± 0.024 | 0.453 ± 0.029, 0.464 ± 0.030 | 0.443 ± 0.027, 0.452 ± 0.027 |
|  | *p value* (aTSA, rTSA) | 0.174, 0.797 | **0.012,** 0.483 | **0.027,** 0.270 |
| **Deltoid Max 2D Diameter, Column (mm)** | Male | 175.3 ± 12.9, 172.3 ± 12.9 | 176.7 ± 12.4, 173.1 ± 13.1 | 174.5 ± 14.3, 173.1 ± 17.1 |
|  | Female | 152.9 ± 11.8, 154.7 ± 12.1 | 157.0 ± 13.3, 153.1 ± 12.5 | 154.1 ± 11.5, 151.2 ± 11.5 |
|  | *p value* (aTSA, rTSA) | **<0.001, <0.001** | **<0.001, <0.001** | **<0.001, <0.001** |
| **Deltoid 10^th^ Percentile, 1^st^**  **Order** | Male | 0.798 ± 0.148, 0.777 ± 0.154 | 0.791 ± 0.165, 0.817 ± 0.142 | 0.813 ± 0.148, 0.817 ± 0.161 |
|  | Female | 0.787 ± 0.128, 0.813 ± 0.141 | 0.804 ± 0.154, 0.827 ± 0.162 | 0.841 ± 0.152, 0.827 ± 0.164 |
|  | *p value* (aTSA, rTSA) | 0.662, 0.185 | 0.467, 0.424 | 0.226, 0.330 |

**Supplemental Table 2**. Comparison of average deltoid muscle radiomic measurements associated with male and female patients, stratified by bone and FC30 convolution kernels

|  | Convolution Kernel/Gender | Male | Female | *p value* |
| --- | --- | --- | --- | --- |
| **Deltoid Normalized Volume** | Bone | 0.99 ± 0.17 | 0.99 ± 0.18 | 0.999 |
|  | FC30 | 1.06 ± 0.20 | 1.05 ± 0.19 | 0.462 |
|  | *p value* | **<0.001** | **<0.001** |  |
| **Deltoid Elongation** | Bone | 0.845 ± 0.060 | 0.773 ± 0.059 | **<0.001** |
|  | FC30 | 0.843 ± 0.062 | 0.764 ± 0.060 | **<0.001** |
|  | *p value* | 0.606 | **0.047** |  |
| **Deltoid Flatness** | Bone | 0.482 ± 0.037 | 0.454 ± 0.040 | **<0.001** |
|  | FC30 | 0.483 ± 0.042 | 0.454 ± 0.043 | **<0.001** |
|  | *p value* | 0.763 | 0.952 |  |
| **Deltoid Fat Percentage (%)** | Bone | 7.4 ± 5.4 | 9.9 ± 6.6 | **<0.001** |
|  | FC30 | 23.1 ± 4.6 | 26.9 ± 4.6 | **<0.001** |
|  | *p value* | **<0.001** | **<0.001** |  |
| **Deltoid Max 2D Diameter, Row (mm)** | Bone | 150.1 ± 17.4 | 124.7 ± 16.4 | **<0.001** |
|  | FC30 | 152.6 ± 15.0 | 124.4 ± 16.5 | **<0.001** |
|  | *p value* | 0.097 | 0.825 |  |
| **Deltoid Sphericity** | Bone | 0.454 ± 0.025 | 0.450 ± 0.025 | **0.018** |
|  | FC30 | 0.462 ± 0.029 | 0.463 ± 0.028 | 0.502 |
|  | *p value* | **0.003** | **<0.001** |  |
| **Deltoid Max 2D Diameter, Column (mm)** | Bone | 173.5 ± 12.9 | 152.8 ± 11.8 | **<0.001** |
|  | FC30 | 174.4 ± 12.3 | 153.2 ± 10.3 | **<0.001** |
|  | *p value* | 0.441 | 0.630 |  |
| **Deltoid 10^th^ Percentile, 1^st^**  **Order** | Bone | 0.836 ± 0.115 | 0.850 ± 0.119 | 0.113 |
|  | FC30 | 0.858 ± 0.083 | 0.858 ± 0.098 | 0.920 |
|  | *p value* | **0.016** | 0.365 |  |

**Supplemental Table 3**. Distribution of deltoid muscle radiomic measurements and patient demographics associated with male and female deltoid clusters

| **Deltoid Cluster** | **Deltoid Normalized Volume** | **Deltoid Elongation** | **Deltoid Flatness** | **Deltoid Fat Percentage** | **Deltoid Max 2D Diameter, Column** | **Deltoid Max 2D Diameter, Row** | **Deltoid Sphericity** | **Deltoid 10^th^ Percentile, 1^st^ Order** | **Patient Age (yrs)** | **Patient BMI** | **Diagnosis OA %** | **Diagnosis RCT %** | **Diagnosis RCT %** | **Prosthesis Type, rTSA %** |
| --- | --- | --- | --- | --- | --- | --- | --- | --- | --- | --- | --- | --- | --- | --- |
| Cluster 0, Male (n=257) | 0.99 ± 0.14 | 0.82 ± 0.06 | 0.50 ± 0.03 | 10.8 ± 10.5 | 180.3 ± 10.7 | 163.2 ± 14.2 | 0.43 ± 0.02 | 0.88 ± 0.14 | 73.6 ± 6.7 | 26.8 ± 3.4 | 62.6% | 17.9% | 30.4% | 80.5% |
| Cluster 1, Male (n=294) | 0.83 ± 0.13 | 0.83 ± 0.06 | 0.47 ± 0.03 | 10.4 ± 10.3 | 163.5 ± 11.2 | 135.4 ± 12.0 | 0.45 ± 0.02 | 0.86 ± 0.14 | 70.9 ± 8.1 | 28.6 ± 4.8 | 69.0% | 16.7% | 23.5% | 78.2% |
| Cluster 2, Male (n=272) | 1.08 ± 0.15 | 0.82 ± 0.05 | 0.45 ± 0.03 | 18.8 ± 10.5 | 182.4 ± 10.9 | 146.9 ± 12.7 | 0.47 ± 0.02 | 0.71 ± 0.15 | 66.3 ± 7.8 | 32.4 ± 5.5 | 80.9% | 14.3% | 15.4% | 61.8% |
| Cluster 3, Male (n=270) | 1.11 ± 0.17 | 0.90 ± 0.04 | 0.50 ± 0.04 | 13.3 ± 10.9 | 169.7 ± 9.4 | 151.7 ± 11.9 | 0.48 ± 0.02 | 0.79 ± 0.14 | 66.1 ± 8 | 31.0 ± 5.0 | 64.4% | 14.8% | 33.0% | 70.4% |
| Cluster 0, Female (n=443) | 0.95 ± 0.15 | 0.79 ± 0.06 | 0.49 ± 0.04 | 9.3 ± 9.0 | 150.2 ± 9.6 | 135.3 ± 13.7 | 0.43 ± 0.02 | 0.93 ± 0.14 | 73.7 ± 7.5 | 24.7 ± 4.1 | 67.7% | 15.1% | 26.4% | 83.3% |
| Cluster 1, Female (n=318) | 1.13 ± 0.18 | 0.75 ± 0.05 | 0.44 ± 0.03 | 18.6 ± 10.9 | 166.4 ± 9.9 | 125.5 ± 15.1 | 0.46 ± 0.02 | 0.75 ± 0.13 | 67.6 ± 7.9 | 32.3 ± 6.2 | 71.7% | 16.7% | 21.1% | 68.6% |
| Cluster 2, Female (n=409) | 0.87 ± 0.13 | 0.73 ± 0.05 | 0.43 ± 0.03 | 15.1 ± 11.1 | 148.7 ± 9.9 | 110.2 ± 10.5 | 0.45 ± 0.02 | 0.84 ± 0.15 | 72.8 ± 7.1 | 29.6 ± 5.6 | 73.8% | 13.9% | 19.1% | 81.2% |
| Cluster 3, Female (n=324) | 1.10 ± 0.16 | 0.82 ± 0.05 | 0.47 ± 0.04 | 21.5 ± 10.8 | 146.2 ± 9.2 | 121.2 ± 11.6 | 0.48 ± 0.02 | 0.74 ± 0.14 | 69.1 ± 8.4 | 32.9 ± 6.7 | 58.3% | 24.1% | 30.6% | 81.8% |

**Supplemental Table 4**. Comparison of deltoid clusters to pre-operative clinical outcomes measures of active range of motion, pain, function, and patient reported outcome measures for male and female patients

| **Deltoid Cluster** | **Active Abduction** | **Active Forward Elevation** | **IR Score** | **Active External Rotation** | **VAS Pain** | **Global Shoulder Function** | **Constant** | **ASES** | **SAS** |
| --- | --- | --- | --- | --- | --- | --- | --- | --- | --- |
| Cluster 0, Male (n=257) | 81.5 ± 36.9 | 97.2 ± 39.2 | 3.4 ± 1.7 | 25.2 ± 22.5 | 5.7 ± 2.2 | 4.4 ± 2.3 | 43.1 ± 15.2 | 42.2 ± 16.1 | 50.5 ± 11.8 |
| Cluster 1, Male (n=294) | 85.2 ± 35.5 | 97.3 ± 36.7 | 3.2 ± 1.8 | 24.0 ± 20.3 | 5.6 ± 2.3 | 4.4 ± 1.8 | 42.5 ± 14.9 | 41.7 ± 15.2 | 49.4 ± 10.7 |
| Cluster 2, Male (n=272) | 94.6 ± 40.2 | 104.2 ± 36.2 | 3.0 ± 1.9 | 25.4 ± 21.3 | 5.8 ± 2.3 | 4.5 ± 2.0 | 42.7 ± 15.5 | 41.6 ± 15.6 | 49.2 ± 12.2 |
| Cluster 3, Male (n=270) | 99.4 ± 39.0 | 112.0 ± 36.7 | 3.5 ± 1.7 | 26.2 ± 21.5 | 5.8 ± 2.2 | 4.7 ± 1.9 | 47.3 ± 15.9 | 42.2 ± 15.4 | 52.4 ± 11.0 |
| *p value* **(**Kruskal-Wallis) | **<0.001** | **<0.001** | **0.019** | 0.580 | 0.933 | 0.342 | **0.011** | 0.840 | **0.005** |
| *p value* **(0M vs 3M)** | **<0.001** | **<0.001** | 0.661 | NA | NA | NA | **0.011** | NA | 0.072 |
| Cluster 0, Female (n=443) | 79.7 ± 34.4 | 97.3 ± 37.1 | 3.5 ± 1.8 | 24.9 ± 21.8 | 6.3 ± 2.2 | 3.9 ± 2.1 | 39.5 ± 14.5 | 37.6 ± 15.7 | 48.2 ± 12.4 |
| Cluster 1, Female (n=318) | 90.4 ± 37.0 | 99.4 ± 35.2 | 3.1 ± 1.8 | 27.5 ± 20.7 | 6.4 ± 2.1 | 4.3 ± 2.0 | 38.7 ± 14.0 | 35.2 ± 15.0 | 47.4 ± 12.3 |
| Cluster 2, Female (n=409) | 75.2 ± 33.7 | 86.2 ± 35.5 | 2.6 ± 1.8 | 19.3 ± 20.0 | 6.7 ± 2.2 | 3.8 ± 2.2 | 34.2 ± 14.2 | 33.0 ± 15.5 | 42.7 ± 12.4 |
| Cluster 3, Female (n=324) | 84.8 ± 40.3 | 96.3 ± 40.8 | 3.1 ± 1.9 | 23.7 ± 21.3 | 6.4 ± 2.1 | 4.1 ± 2.2 | 35.9 ± 14.7 | 33.9 ± 14.7 | 46.4 ± 12.8 |
| *p value* **(**Kruskal-Wallis) | **<0.001** | **<0.001** | **<0.001** | **<0.001** | **0.025** | **0.005** | **<0.001** | **<0.001** | **<0.001** |
| *p value* **(1F vs 2F)** | **<0.001** | **<0.001** | **<0.001** | **<0.001** | **0.049** | **0.002** | **<0.001** | 0.073 | **<0.001** |

**Supplemental Table 5**. Comparison of deltoid clusters to 2-year minimum clinical outcomes measures of active range of motion, pain, function, and patient reported outcome measures for male and female patients

| **Deltoid Cluster** | **Active Abduction** | **Active Forward Elevation** | **IR Score** | **Active External Rotation** | **VAS Pain** | **Global Shoulder Function** | **Constant** | **ASES** | **SAS** |
| --- | --- | --- | --- | --- | --- | --- | --- | --- | --- |
| Cluster 0, Male (n=257) | 128.9 ± 26.0 | 145.2 ± 21.2 | 4.3 ± 1.7 | 43.7 ± 18.2 | 1.1 ± 2.1 | 8.5 ± 1.9 | 71.9 ± 13.5 | 84.6 ± 17.6 | 75.1 ± 11.4 |
| Cluster 1, Male (n=294) | 126.8 ± 31.6 | 145.0 ± 23.3 | 3.9 ± 1.7 | 42.1 ± 20.7 | 1.3 ± 2.0 | 8.2 ± 2.1 | 69.5 ± 14.6 | 83.6 ± 18.0 | 74.1 ± 12.4 |
| Cluster 2, Male (n=272) | 145.1 ± 23.3 | 151.3 ± 18.3 | 4.7 ± 1.4 | 50.5 ± 18.3 | 1.2 ± 2.2 | 8.6 ± 1.7 | 75.0 ± 12.1 | 86.2 ± 17.8 | 78.4 ± 10.3 |
| Cluster 3, Male (n=270) | 134.9 ± 25.1 | 149.9 ± 17.4 | 4.4 ± 1.7 | 47.0 ± 18.8 | 1.4 ± 2.3 | 8.3 ± 2.1 | 73.8 ± 13.5 | 84.0 ± 19.0 | 75.5 ± 11.8 |
| *p value* **(**Kruskal-Wallis) | **<0.001** | 0.202 | **0.016** | **0.012** | 0.418 | 0.626 | 0.061 | 0.652 | 0.104 |
| *p value* **(1M vs 2M)** | **<0.001** | NA | **<0.001** | **0.003** | NA | NA | NA | NA | NA |
| Cluster 0, Female (n=443) | 125.1 ± 27.7 | 148.2 ± 23.4 | 5.0 ± 1.4 | 44.2 ± 20.0 | 1.3 ± 2.1 | 8.5 ± 1.9 | 70.1 ± 12.8 | 84.8 ± 17.9 | 77.5 ± 12.2 |
| Cluster 1, Female (n=318) | 131.3 ± 32.5 | 143.1 ± 27.0 | 4.7 ± 1.5 | 48.2 ± 17.2 | 1.4 ± 2.3 | 8.2 ± 2.1 | 68.2 ± 14.1 | 81.8 ± 19.6 | 75.4 ± 11.9 |
| Cluster 2, Female (n=409) | 128.3 ± 30.8 | 142.6 ± 27.9 | 4.6 ± 1.6 | 44.8 ± 17.9 | 1.0 ± 1.9 | 8.5 ± 1.8 | 66.6 ± 14.8 | 84.9 ± 16.3 | 75.5 ± 12.1 |
| Cluster 3, Female (n=324) | 129.1 ± 34.8 | 140.6 ± 29.2 | 4.2 ± 1.8 | 45.5 ± 20.2 | 1.4 ± 2.1 | 7.9 ± 2.1 | 63.6 ± 14.7 | 80.1 ± 18.7 | 72.6 ± 13.0 |
| *p value* **(**Kruskal-Wallis) | 0.091 | **0.039** | **<0.001** | 0.099 | 0.265 | **0.004** | **0.001** | **0.026** | **0.003** |
| *p value* **(0F vs 3F)** | NA | **0.010** | **<0.001** | NA | NA | **0.003** | **<0.001** | **0.015** | **<0.001** |

**Supplemental Table 6**. Comparison of average scapular bone radiomic measurements associated with primary aTSA and rTSA patients, stratified by gender and age at the time of surgery

| **aTSA, rTSA** | **Age at time of surgery (yrs)** | **<60** | **60-70** | **70-80** |
| --- | --- | --- | --- | --- |
| **Scapula Elongation** | Male | 0.513 ± 0.032, 0.510 ± 0.038 | 0.507 ± 0.034, 0.504 ± 0.035 | 0.501 ± 0.030, 0.501 ± 0.034 |
|  | Female | 0.514 ± 0.033, 0.517 ± 0.031 | 0.520 ± 0.037, 0.514 ± 0.033 | 0.515 ± 0.031, 0.516 ± 0.035 |
|  | *p value* (aTSA, rTSA) | 0.826, 0.187 | **<0.001, <0.001** | **<0.001, <0.001** |
| **Scapula Flatness** | Male | 0.293 ± 0.026, 0.299 ± 0.040 | 0.295 ± 0.026, 0.301 ± 0.031 | 0.298 ± 0.025, 0.299 ± 0.026 |
|  | Female | 0.283 ± 0.026, 0.292 ± 0.026 | 0.295 ± 0.036, 0.293 ± 0.026 | 0.291 ± 0.022, 0.297 ± 0.027 |
|  | *p value* (aTSA, rTSA) | **0.011,** 0.232 | 0.968, <**0.001** | **0.034,** 0.108 |
| **Scapula Max 2D Diameter, Column (mm)** | Male | 122.2 ± 11.7, 125.6 ± 11.3 | 121.8 ± 10.4, 125.2 ± 12.6 | 121.2 ± 8.9, 125.0 ± 13.1 |
|  | Female | 105.0 ± 11.9, 108.6 ± 11.2 | 104.3 ± 8.7, 105.7 ± 11.2 | 101.8 ± 9.0, 104.7 ± 9.9 |
|  | *p value* (aTSA, rTSA) | **<0.001, <0.001** | **<0.001, <0.001** | **<0.001, <0.001** |
| **Scapula Sphericity** | Male | 0.236 ± 0.014, 0.236 ± 0.016 | 0.237 ± 0.013, 0.235 ± 0.014 | 0.236 ± 0.013, 0.233 ± 0.013 |
|  | Female | 0.238 ± 0.013, 0.234 ± 0.015 | 0.235 ± 0.016, 0.230 ± 0.014 | 0.234 ± 0.014, 0.230 ± 0.014 |
|  | *p value* (aTSA, rTSA) | 0.524, 0.430 | 0.173, **<0.001** | 0.264, <**0.001** |
| **Scapula, 10^th^ Percentile, 1^st^ Order** | Male | 1.002 ± 0.185, 0.955 ± 0.184 | 0.968 ± 0.184, 0.966 ± 0.170 | 0.981 ± 0.184, 0.956 ± 0.164 |
|  | Female | 1.043 ± 0.168, 1.029 ± 0.229 | 0.989 ± 0.179, 1.002 ± 0.178 | 1.001 ± 0.189, 0.987 ± 0.184 |
|  | *p value* (aTSA, rTSA) | 0.126, **0.032** | 0.249, **0.006** | 0.420, **0.002** |
| **Scapula Max 2D Diameter, Row (mm)** | Male | 163.2 ± 10.1, 164.5 ± 18.1 | 164.2 ± 11.2, 165.1 ± 14.5 | 162.6 ± 10.8, 165.6 ± 13.2 |
|  | Female | 140.9 ± 14.7, 145.8 ± 10.2 | 139.5 ± 9.6, 144.8 ± 12.3 | 141.9 ± 10.0, 142.5 ± 11.5 |
|  | *p value* (aTSA, rTSA) | **<0.001, <0.001** | **<0.001, <0.001** | **<0.001, <0.001** |
| **Scapula Least Axis Length (mm)** | Male | 57.4 ± 4.8, 57.8 ± 5.2 | 58.1 ± 4.3, 59.0 ± 4.5 | 58.5 ± 4.0, 58.9 ± 4.2 |
|  | Female | 47.8 ± 3.7, 50.1 ± 4.3 | 49.4 ± 3.8, 50.1 ± 3.9 | 49.2 ± 3.7, 49.9 ± 3.5 |
|  | *p value* (aTSA, rTSA) | **<0.001, <0.001** | **<0.001, <0.001** | **<0.001, <0.001** |
| **Scapula Max 2D Diameter, Slice (mm)** | Male | 142.7 ± 11.5, 148.3 ± 14.9 | 144.0 ± 11.2, 145.5 ± 12.0 | 142.2 ± 9.7, 141.7 ± 10.5 |
|  | Female | 121.6 ± 10.1, 125.1 ± 11.3 | 125.6 ± 9.8, 121.8 ± 11.1 | 124.4 ± 9.3, 118.4 ± 9.6 |
|  | *p value* (aTSA, rTSA) | **<0.001, <0.001** | **<0.001, <0.001** | **<0.001, <0.001** |

**Supplemental Table 7**. Comparison of average scapular bone radiomic measurements associated with male and female patients, stratified by bone and FC30 convolution kernels

|  | **Convolution Kernel/Gender** | **Male** | **Female** | ***p value*** |
| --- | --- | --- | --- | --- |
| **Scapula Elongation** | Bone | 0.500 ± 0.035 | 0.512 ± 0.033 | **<0.001** |
|  | FC30 | 0.500 ± 0.034 | 0.515 ± 0.031 | **<0.001** |
|  | *p value* | 0.862 | 0.151 |  |
| **Scapula Flatness** | Bone | 0.299 ± 0.029 | 0.296 ± 0.023 | 0.132 |
|  | FC30 | 0.297 ± 0.025 | 0.295 ± 0.023 | 0.394 |
|  | *p value* | 0.303 | 0.383 |  |
| **Scapula Max 2D Diameter, Column (mm)** | Bone | 124.4 ± 12.7 | 104.2 ± 9.7 | **<0.001** |
|  | FC30 | 124.8 ± 10.9 | 105.8 ± 9.5 | **<0.001** |
|  | *p value* | 0.694 | **0.023** |  |
| **Scapula Sphericity** | Bone | 0.234 ± 0.013 | 0.229 ± 0.013 | **<0.001** |
|  | FC30 | 0.234 ± 0.013 | 0.231 ± 0.013 | **0.013** |
|  | *p value* | 0.771 | **0.029** |  |
| **Scapula, 10^th^ Percentile, 1^st^ Order** | Bone | 0.945 ± 0.135 | 0.965 ± 0.150 | **0.025** |
|  | FC30 | 1.002 ± 0.093 | 0.997 ± 0.104 | 0.502 |
|  | *p value* | **<0.001** | **0.002** |  |
| **Scapula Max 2D Diameter, Row** | Bone | 165.2 ± 13.0 | 143.1 ± 11.1 | **<0.001** |
|  | FC30 | 163.4 ± 11.7 | 141.9 ± 10.0 | **<0.001** |
|  | *p value* | 0.064 | 0.136 |  |
| **Scapula Least Axis Length (mm)** | Bone | 58.9 ± 4.1 | 50.3 ± 3.4 | **<0.001** |
|  | FC30 | 58.6 ± 4.7 | 49.9 ± 3.7 | **<0.001** |
|  | *p value* | 0.337 | 0.077 |  |
| **Scapula Max 2D Diameter, Slice (mm)** | Bone | 140.0 ± 10.6 | 125.7 ± 10.6 | **<0.001** |
|  | FC30 | 138.3 ± 10.9 | 122.2 ± 11.0 | **<0.001** |
|  | *p value* | **0.038** | **<0.001** |  |

**Supplemental Table 8**. Distribution of Scapular Bone Radiomic Measurements and Patient Demographics Associated with Male and Female Deltoid Clusters

| **Scapula Cluster** | **Scapula Elongation** | **Scapula Flatness** | **Scapula Least Axis Length (mm)** | **Scapula Max 2D Diameter, Column (mm)** | **Scapula Max 2D Diameter, Row (mm)** | **Scapula Max 2D Diameter, Slice (mm)** | **Scapula Sphericity** | **Scapula 10^th^ Percentile, 1^st^ Order** | **Patient Age (yrs)** | **Patient BMI** | **Diagnosis OA %** | **Diagnosis RCT %** | **Diagnosis RCT %** | **Prosthesis Type, rTSA %** |
| --- | --- | --- | --- | --- | --- | --- | --- | --- | --- | --- | --- | --- | --- | --- |
| Cluster 0, Male (n=579) | 0.50 ± 0.03 | 0.28 ± 0.02 | 54.8 ± 2.9 | 118.3 ± 8.7 | 158.2 ± 9.6 | 135.5 ± 9.2 | 0.24 ± 0.01 | 1.01 ± 0.17 | 66.5 ± 9.1 | 29.5 ± 5.3 | 75.3% | 13.1% | 18.8% | 60.8% |
| Cluster 1, Male (n=218) | 0.50 ± 0.03 | 0.30 ± 0.02 | 59.9 ± 4.2 | 143.5 ± 12.1 | 180.5 ± 13.3 | 131.1 ± 9.9 | 0.23 ± 0.01 | 0.93 ± 0.18 | 71.1 ± 8.1 | 30.3 ± 6.0 | 50.0% | 22.0% | 45.4% | 86.7% |
| Cluster 2, Male (n=549) | 0.49 ± 0.02 | 0.30 ± 0.02 | 60.3 ± 3.0 | 121.9 ± 8.1 | 166.9 ± 9.7 | 146.1 ± 9.8 | 0.23 ± 0.01 | 0.94 ± 0.17 | 70.8 ± 7.6 | 29.4 ± 4.7 | 69.8% | 13.7% | 25.7% | 74.7% |
| Cluster 3, Male (n=267) | 0.54 ± 0.04 | 0.33 ± 0.04 | 62.6 ± 3.6 | 124.7 ± 8.8 | 161.0 ± 13.6 | 146.2 ± 10.6 | 0.24 ± 0.01 | 0.96 ± 0.17 | 65.7 ± 8.0 | 31.2 ± 5.7 | 79.4% | 11.6% | 18.4% | 58.1% |
| Cluster 0, Female (n=511) | 0.50 ± 0.03 | 0.29 ± 0.02 | 50.2 ± 2.4 | 100.2 ± 7.0 | 141.9 ± 9.5 | 129.9 ± 9.2 | 0.23 ± 0.01 | 0.97 ± 0.16 | 74.7 ± 6.2 | 28.5 ± 6.1 | 67.9% | 16.6% | 24.3% | 85.5% |
| Cluster 1, Female (n=363) | 0.54 ± 0.04 | 0.33 ± 0.03 | 52.8 ± 3.0 | 104.9 ± 7.4 | 140.8 ± 11.2 | 126.3 ± 9.7 | 0.24 ± 0.01 | 0.95 ± 0.17 | 70.8 ± 7.3 | 30.5 ± 6.6 | 74.4% | 18.2% | 20.1% | 78.2% |
| Cluster 2, Female (n=318) | 0.51 ± 0.03 | 0.29 ± 0.02 | 51.9 ± 3.1 | 117.7 ± 10.1 | 154.9 ± 11.8 | 123.0 ± 11.2 | 0.22 ± 0.01 | 1.00 ± 0.19 | 69.9 ± 8.6 | 30.0 ± 7.7 | 60.1% | 18.2% | 34.6% | 88.1% |
| Cluster 3, Female (n=535) | 0.51 ± 0.03 | 0.28 ± 0.02 | 46.3 ± 2.4 | 101.5 ± 8.4 | 137.8 ± 9.2 | 118.1 ± 9.1 | 0.24 ± 0.01 | 1.05 ± 0.19 | 68.5 ± 8.1 | 29.8 ± 7.1 | 73.3% | 15.3% | 19.1% | 67.3% |

**Supplemental Table 9**. Comparison of Scapula Clusters to Pre-operative Clinical Outcomes Measures of Active Range of Motion, Pain, Function, and Patient Reported Outcome Measures for Male and Female Patients

| **Scapula Cluster** | **Active Abduction** | **Active Forward Elevation** | **IR Score** | **Active External Rotation** | **VAS Pain** | **Global Shoulder Function** | **Constant** | **ASES** | **SAS** |
| --- | --- | --- | --- | --- | --- | --- | --- | --- | --- |
| Cluster 0, Male (n=579) | 94.3 ± 36.4 | 107.0 ± 35.3 | 3.5 ± 1.7 | 26.2 ± 21.2 | 5.9 ± 2.2 | 4.6 ± 2.0 | 47.3 ± 15.4 | 41.8 ± 15.8 | 51.4 ± 11.3 |
| Cluster 1, Male (n=218) | 88.3 ± 40.9 | 97.9 ± 41.4 | 3.6 ± 1.9 | 26.2 ± 22.1 | 5.5 ± 2.3 | 4.2 ± 2.0 | 42.7 ± 15.9 | 43.1 ± 16.8 | 50.9 ± 11.8 |
| Cluster 2, Male (n=549) | 91.2 ± 39.2 | 103.4 ± 37.4 | 3.3 ± 1.7 | 23.5 ± 21.5 | 5.5 ± 2.3 | 4.6 ± 2.1 | 44.7 ± 15.5 | 43.4 ± 16.6 | 50.5 ± 11.5 |
| Cluster 3, Male (n=267) | 92.5 ± 35.9 | 107.0 ± 35.2 | 3.1 ± 1.7 | 22.4 ± 22.7 | 5.8 ± 2.2 | 4.6 ± 1.8 | 45.0 ± 15.2 | 41.0 ± 15.7 | 49.6 ± 11.2 |
| *p value* **(**Kruskal-Wallis) | 0.097 | **0.018** | **0.004** | 0.085 | 0.083 | **0.041** | **0.010** | 0.222 | 0.230 |
| *p value* **(0M vs 1M)** | **NA** | **0.002** | 0.569 | NA | NA | **0.016** | **0.003** | NA | **NA** |
| Cluster 0, Female (n=511) | 78.8 ± 33.2 | 93.8 ± 34.8 | 3.0 ± 1.8 | 20.3 ± 21.2 | 6.1 ± 2.2 | 4.2 ± 2.1 | 38.2 ± 13.4 | 37.3 ± 15.7 | 46.3 ± 11.8 |
| Cluster 1, Female (n=363) | 82.9 ± 37 | 95.2 ± 36.4 | 3.0 ± 1.9 | 24.1 ± 21.9 | 6.6 ± 2.1 | 4 ± 2.1 | 37.8 ± 14.4 | 34.5 ± 15.3 | 46.1 ± 12.4 |
| Cluster 2, Female (n=318) | 79.9 ± 39.4 | 91.7 ± 39.8 | 3.3 ± 1.9 | 25.2 ± 22.9 | 6.3 ± 2.2 | 3.9 ± 2.1 | 35.8 ± 15.2 | 35.5 ± 15.9 | 46.6 ± 13.6 |
| Cluster 3, Female (n=535) | 85.6 ± 35.9 | 96.2 ± 36.2 | 3.1 ± 1.9 | 24.2 ± 20.5 | 6.7 ± 2.1 | 4.2 ± 2.1 | 37.3 ± 14.6 | 33.8 ± 15.6 | 45.8 ± 12.9 |
| *p value* **(**Kruskal-Wallis) | **0.005** | 0.176 | 0.378 | **0.005** | **<0.001** | 0.194 | 0.173 | **0.006** | 0.865 |
| *p value* **(0F vs 3F)** | **0.002** | **NA** | **NA** | **0.003** | **<0.001** | **NA** | **NA** | **<0.001** | **NA** |

**Supplemental Table 10**. Comparison of Scapula Clusters to 2-Year Minimum Clinical Outcomes Measures of Active Range of Motion, Pain, Function, and Patient Reported Outcome Measures for Male and Female Patients

| **Scapula Cluster** | **Active Abduction** | **Active Forward Elevation** | **IR Score** | **Active External Rotation** | **VAS Pain** | **Global Shoulder Function** | **Constant** | **ASES** | **SAS** |
| --- | --- | --- | --- | --- | --- | --- | --- | --- | --- |
| Cluster 0, Male (n=579) | 132.2 ± 28.9 | 148.0 ± 24.0 | 4.5 ± 1.6 | 47.3 ± 18.4 | 1.1 ± 1.9 | 8.6 ± 1.8 | 72.3 ± 15.1 | 86.3 ± 16.7 | 77.5 ± 11.8 |
| Cluster 1, Male (n=218) | 130.9 ± 26.8 | 142.6 ± 24.8 | 4.2 ± 1.7 | 42.4 ± 19.9 | 1.4 ± 2.1 | 8.2 ± 2.0 | 69.8 ± 16.6 | 81.8 ± 20.0 | 74.7 ± 12.2 |
| Cluster 2, Male (n=549) | 134.6 ± 27.9 | 148.3 ± 22.2 | 4.1 ± 1.6 | 44.5 ± 17.9 | 1.2 ± 2.1 | 8.4 ± 1.8 | 71.9 ± 12.9 | 85.7 ± 17.0 | 76.8 ± 10.4 |
| Cluster 3, Male (n=267) | 138.7 ± 31.0 | 148.6 ± 27.0 | 4.3 ± 1.7 | 47.7 ± 20.0 | 0.9 ± 1.8 | 8.7 ± 1.8 | 71.8 ± 14.7 | 87.7 ± 15.9 | 78.5 ± 11.8 |
| *p value* **(**Kruskal-Wallis) | 0.062 | 0.094 | 0.126 | 0.219 | 0.309 | 0.119 | 0.834 | 0.107 | 0.055 |
| Cluster 0, Female (n=511) | 128.2 ± 29.8 | 144.9 ± 25.6 | 4.6 ± 1.6 | 42.1 ± 20.1 | 1.1 ± 2.0 | 8.4 ± 2.0 | 67.1 ± 13.8 | 84.1 ± 17.1 | 76.6 ± 11.7 |
| Cluster 1, Female (n=363) | 127.8 ± 30.9 | 141.6 ± 27.8 | 4.6 ± 1.6 | 45.1 ± 18.5 | 1.0 ± 1.8 | 8.5 ± 1.8 | 66.5 ± 13.4 | 84.6 ± 16.1 | 76.4 ± 11.1 |
| Cluster 2, Female (n=318) | 124.0 ± 30.1 | 140.9 ± 27.1 | 4.6 ± 1.8 | 45.6 ± 18.7 | 1.3 ± 2.0 | 8.2 ± 1.9 | 68.5 ± 13.4 | 81.9 ± 18.0 | 76.0 ± 12.7 |
| Cluster 3, Female (n=535) | 129.4 ± 32.9 | 143.6 ± 28.1 | 4.7 ± 1.5 | 47.7 ± 18.7 | 1.4 ± 2.3 | 8.2 ± 2.2 | 66.7 ± 14.9 | 82.2 ± 19.4 | 75.8 ± 12.3 |
| *p value* **(**Kruskal-Wallis) | 0.186 | 0.310 | 0.738 | **0.011** | 0.137 | 0.221 | 0.693 | 0.241 | 0.934 |
| *p value* **(0F vs 3F)** | NA | NA | NA | **<0.001** | NA | NA | NA | NA | NA |

**Supplementary Table 11**. Definitions of radiomic feature measurements and domain measurements

| **Radiomic Feature** | **Radiomic Feature Type** | **Feature Definition** |
| --- | --- | --- |
| Elongation | Shape | Shows the relationship between the two largest principal components in the ROI shape. The values range between 1 (where the cross section through the first and second largest principal moments is circle-like (non-elongated)) and 0 (where the object is a maximally elongated: i.e. a 1 dimensional line). |
| Flatness | Shape | Shows the relationship between the largest and smallest principal components in the ROI shape. The values range between 1 (non-flat, sphere-like) and 0 (a flat object, or single-slice segmentation). |
| Least Axis Length | Shape | Yields the smallest axis length of the ROI-enclosing ellipsoid and is calculated using the largest principal component λ_least._ |
| Major Axis Length | Shape | Yields the largest axis length of the ROI-enclosing ellipsoid and is calculated using the largest principal component λ_major._ |
| Minor Axis Length | Shape | Yields the medium (2^nd^ largest) axis length of the ROI-enclosing ellipsoid and is calculated using the largest principal component λ_minor_. |
| Max 2D Diameter, Column | Shape | Maximum 2D diameter (Column) is defined as the largest pairwise Euclidean distance between surface mesh vertices in the row-slice (usually the coronal) plane. |
| Max 2D Diameter, Row | Shape | Maximum 2D diameter (Row) is defined as the largest pairwise Euclidean distance between surface mesh vertices in the column-slice (usually the sagittal) plane. |
| Max 2D Diameter, Slice | Shape | Maximum 2D diameter (Slice) is defined as the largest pairwise Euclidean distance between surface mesh vertices in the row-column (generally the axial) plane. |
| Mesh Volume | Shape | Volume of the RIO calculated from the triangle mesh of the ROI. |
| Sphericity | Shape | A measure of the roundness of the shape of the ROI relative to a sphere. It is a dimensionless measure, independent of scale and orientation. The value range is 0 < sphericity ≤ 1, where a value of 1 indicates a perfect sphere. |
| Surface to Volume Ratio | Shape | Surface area divided by volume, where a lower value indicates a more compact (sphere-like) shape. |
| 10^th^ Percentile | First-Order | 10^th^ percentile voxel intensity. |
| Entropy | First-Order | Entropy specifies the uncertainty/randomness in the image values. It measures the average amount of information required to encode the image values. |
| Interquartile Range | First-Order | 75^th^ percentile grey level intensity minus the 25^th^ percentile grey level intensity. |
| Range | First-Order | Range of grey level intensities. |
| Kurtosis | First-Order | A measure of the ‘peakedness’ of the distribution of values in the image ROI. A higher kurtosis implies that the mass of the distribution is concentrated towards the tail(s) rather than towards the mean. A lower kurtosis implies the reverse: that the mass of the distribution is concentrated towards a spike near the Mean value. |
| Maximum | First-Order | Maximum grey level intensity. |
| Mean | First-Order | Mean grey level intensity. |
| Minimum | First-Order | Minimum grey level intensity. |
| Skewness | First-Order | Measures the asymmetry of the distribution of values about the mean value. Depending on where the tail is elongated and the mass of the distribution is concentrated, this value can be positive or negative. |
| Autocorrelation | Second-Order/Texture (GLCM) | A measure of the magnitude of the fineness and coarseness of texture. |
| Cluster Tendency | Second-Order/Texture (GLCM) | A measure of groupings of voxels with similar gray-level values. |
| Contrast | Second-Order/Texture (GLCM) | A measure of the local intensity variation, favoring values away from the diagonal (i=j). A larger value correlates with a greater disparity in intensity values among neighboring voxels. |
| Correlation | Second-Order/Texture (GLCM) | A value between 0 (uncorrelated) and 1 (perfectly correlated) showing the linear dependency of gray level values to their respective voxels in the GLCM. |
| Id | Second-Order/Texture (GLCM) | A measure of the local homogeneity of an image. With more uniform gray levels, the denominator will remain low, resulting in a higher overall value. |
| Joint Energy | Second-Order/Texture (GLCM) | A measure of homogeneous patterns in the image. A greater Energy implies that there are more instances of intensity value pairs in the image that neighbor each other at higher frequencies. |
| Joint Entropy | Second-Order/Texture (GLCM) | A measure of the randomness/variability in neighborhood intensity values. |
| Maximum Probability | Second-Order/Texture (GLCM) | A measure of occurrences of the most predominant pair of neighboring intensity values. |
| Maximal Correlation Coefficient (MCC) | Second-Order/Texture (GLCM) | A measure of complexity of the texture and 0≤MCC≤1. |
| Sum Entropy | Second-Order/Texture (GLCM) | Specifies the uncertainty/randomness in the image values. |
| Dependence Entropy | Second-Order/Texture (GLDM) | 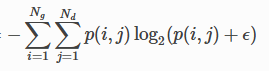 |
| Dependence Variance | Second-Order/Texture (GLDM) | A measure of the variance in dependence size in the image. |
| Gray Level Variance | Second-Order/Texture (GLDM) | A measure of the variance in gray level intensities for the zones. |
| Large Dependence High Gray Level Emphasis | Second-Order/Texture (GLDM) | A measure of the joint distribution of large dependence with higher gray-level values. |
| Large Dependence Low Gray Level Emphasis | Second-Order/Texture (GLDM) | A measure of the joint distribution of large dependence with lower gray-level values. |
| Low Gray Level Emphasis | Second-Order/Texture (GLDM) | A measure of the distribution of low gray-level values, with a higher value indicating a greater concentration of low gray-level values in the image. |
| Small Dependence Low Gray Level Emphasis | Second-Order/Texture (GLDM) | A measure of the joint distribution of small dependence with lower gray-level values. |
| Run Entropy | Second-Order/Texture (GLRLM) | A measure of the uncertainty/randomness in the distribution of run lengths and gray levels. A higher value indicates more heterogeneity in the texture patterns. |
| Gray Level Non-Uniformity Normalized | Second-Order/Texture (GLSZM) | A measure of the variability of gray-level intensity values in the image, with a lower value indicating a greater similarity in intensity values. |
| Gray Level Variance | Second-Order/Texture (GLSZM) | A measure of the variance in grey level in the image. |
| Size-Zone Non-Uniformity Normalized | Second-Order/Texture (GLSZM) | A measure of the variability of size zone volumes in the image, with a lower value indicating more homogeneity in size zone volumes. |
| Low Gray Level Zone Emphasis | Second-Order/Texture (GLSZM) | A measure of the distribution of lower gray-level size zones, with a higher value indicating a greater proportion of lower gray-level values and size zones in the image. |
| Zone Entropy | Second-Order/Texture (GLSZM) | A measure of the uncertainty/randomness in the distribution of zone sizes and gray levels. A higher value indicates more heterogeneity in the texture patterns. |
| Courseness | Second-Order/Texture (NGTDM) | A measure of average difference between the center voxel and its neighborhood and is an indication of the spatial rate of change. A higher value indicates a lower spatial change rate and a locally more uniform texture. |
| Strength | Second-Order/Texture (NGTDM) | A measure of the primitives in an image. Its value is high when the primitives are easily defined and visible, i.e. an image with slow change in intensity but more large coarse differences in gray level intensities. |
| Deltoid Fat Percentage | Domain | Deltoid fat percentage was defined as the number of voxels in the deltoid representing fat (HU between -190 and -30) divided by the number of voxels representing both muscle and fat (HU between -190 and 150). |
| Deltoid Normalized Volume | Domain | Deltoid normalized volume was defined as the volume of a deltoid divided by the average deltoid volume for an age- and gender-matched population. |
